# Increased production of piRNAs from euchromatic clusters and genes in *Anopheles gambiae* compared with *Drosophilamelanogaster*

**DOI:** 10.1186/s13072-015-0041-5

**Published:** 2015-11-27

**Authors:** Phillip George, Silke Jensen, Romain Pogorelcnik, Jiyoung Lee, Yi Xing, Emilie Brasset, Chantal Vaury, Igor V. Sharakhov

**Affiliations:** Department of Entomology, Virginia Polytechnic Institute and State University, Blacksburg, VA 24061 USA; Laboratoire Génétique, Reproduction, et Développement, Clermont Université, Université d’Auvergne, BP 38, 63001 Clermont-Ferrand, France; Institut National de la Santé et de la Recherche Médicale, U 1103, BP 38, 63001 Clermont-Ferrand, France; Centre National de Recherche Scientifique, UMR 6293, BP 38, 63001 Clermont-Ferrand, France; The PhD Program in Genomics Bioinformatics and Computational Biology, Virginia Polytechnic Institute and State University, Blacksburg, VA 24061 USA

**Keywords:** *Anopheles gambiae*, Development, *Drosophila melanogaster*, Euchromatin, Germline, Heterochromatin, piRNA clusters, Reproduction, Small RNAs, Transposable element

## Abstract

**Background:**

Specific genomic loci, termed Piwi-interacting RNA (piRNA) clusters, manufacture piRNAs that serve as guides for the inactivation of complementary transposable elements (TEs). The piRNA pathway has been accurately detailed in *Drosophila melanogaster*, while it remains poorly examined in other insects. This pathway is increasingly recognized as critical for germline development and reproduction. Understanding of the piRNA functions in mosquitoes could offer an opportunity for disease vector control by the reduction of their reproductive potential.

**Results:**

To analyze the similarities and differences in this pathway between *Drosophila* and mosquito, we performed an in-depth analysis of the genomic loci producing piRNAs and their targets in the African malaria vector *Anopheles gambiae*. We identified 187 piRNA clusters in the *An. gambiae* genome and 155 piRNA clusters in the *D. melanogaster* genome. We demonstrate that many more piRNA clusters in the mosquito compared with the fruit fly are uni-directionally transcribed and are located outside pericentromeric heterochromatin. About 11 % of the *An. gambiae* piRNA population map to gene transcripts. This is a noticeable increase compared with the ~6 % of the piRNA population mapped to genes in *D. melanogaster*. A subset of the piRNA-enriched genes in *An. gambiae* has functions related to reproduction and development. At least 24 and 65 % of the mapped piRNAs correspond to genomic TE sequences in *An. gambiae* and *D. melanogaster*, respectively. DNA transposons and non-LTR retrotransposons are more abundant in *An. gambiae*, while LTR retrotransposons are more abundant in *D. melanogaster*. Yet, piRNAs predominantly target LTR retrotransposons in both species, which may point to a distinct feature of these elements compared to the other classes of TEs concerning their silencing by the piRNA pathway.

**Conclusions:**

Here, we demonstrate that piRNA-producing loci have more ubiquitous distribution in the *An. gambiae* genome than in the genome of *D. melanogaster.* Also, protein-coding genes have an increased role in production of piRNAs in the germline of this mosquito. Genes involved in germline and embryonic development of *An. gambiae* generate a substantial portion of piRNAs, suggesting a role of the piRNA pathway in the epigenetic regulation of the reproductive processes in the African malaria vector.

**Electronic supplementary material:**

The online version of this article (doi:10.1186/s13072-015-0041-5) contains supplementary material, which is available to authorized users.

## Background

Piwi-interacting RNAs (piRNAs) are 24–30 nucleotide (nt) small RNAs that play an important role in silencing active transposable elements (TEs) through slicer-mediated cleavage of messenger RNA (mRNA) [1]. piRNAs are by far the most numerous among all types of coding and non-coding RNAs in any animal, mostly acting in the germline. piRNAs of different species share similar features, including a typical motif of predominant Uridine at position one (1U) of antisense TE-derived piRNAs and Adenine at position ten (10A) of sense strand TE-derived piRNAs [2]. Members of the PIWI clade, a subfamily of Argonaute, interact with piRNAs to effectively create an RNA-induced silencing complex (RISC) that can target and silence complementary TE mRNA sequences. A mutation of any of the three key PIWI proteins—Piwi, Aubergine (Aub), and Argonaute 3 (Ago3)—results in de-repression of TEs with mutagenic or disruptive consequences in the *Drosophila melanogaster* germline [3–5], indicating the necessity of these proteins in functional TE silencing. Two mechanisms for piRNA production have been identified in *D. melanogaster* [2]; both mechanisms stem from long single-stranded piRNA precursors that originate from vestigial TEs. In the first mechanism, single-stranded RNA transcripts are processed into primary piRNAs, which are loaded onto the Piwi protein. This process has been referred to as primary piRNA biogenesis [2]. Trimming of the piRNA to the 24–30 nt characteristic size of these small RNAs requires the cytoplasmic endonuclease Zucchini [6, 7]. In another mechanism, secondary piRNAs, responsible for a large portion of the total piRNA pool in the germline, are generated through an amplification loop referred to as the ping-pong cycle [2] and loaded onto Ago3. The Aub protein is posited to work within the ping-pong cycle by binding tertiary piRNAs that are generated through the amplification loop. A ten base-pair overlap can be seen between complementary primary and secondary piRNAs [2, 8]. Many piRNAs associating with the Aubergine and Piwi proteins are antisense to TEs and show a typical 1U feature, while piRNAs associated with Argonaute 3 are sense to the TE transcripts and show a 10A feature.

The piRNA pathway is a major epigenetic programming mechanism in higher eukaryotes and it has been increasingly implicated in germline development of eukaryotes. The Piwi protein is essential to fertility in *D. melanogaster* [9, 10], *Caenorhabditis elegans* [11, 12]*, Danio rerio* [13]*, and Mus musculus* [14]. Germline stem cell loss has also been documented in multiple organisms as a result of piRNA pathway mutation [9, 10, 14–17]. The PIWI proteins of the Asian malaria vector *Anopheles stephensi* are expressed at high levels in the germline cells of ovaries as expected and, importantly, their expression is further increased after a blood meal [18]. In addition to TE-derived piRNAs, a fraction of piRNAs map in the sense orientation to the 3′ untranslated regions (UTRs) of protein-coding transcripts [19–23]. piRNAs are produced by various protein-coding genes, including *stellate, suppressor of stellate* [24], and *traffic jam* [19, 25], that are important for the germline development in *D. melanogaster*. piRNAs from *suppressor of stellate* functionally silence *stellate* transcripts, and a deletion of *suppressor of stellate* leads to *stellate* overexpression and meiotic abnormalities in *Drosophila* testis [24]. *Wolbachia* can control the maternal transmission of endogenous gypsy retroviruses in *D. melanogaster* [26]. Knowledge of the mechanisms of how the piRNA pathway regulates reproduction in mosquitoes could be useful for both basic and applied science. Our increased understanding of reproductive processes in disease vectors will facilitate the identification of novel targets for vector control [27]. The piRNA pathway has also been linked to other epidemiologically important phenotypes in mosquitoes. For example, a role of piRNAs in antiviral immune responses in both *Aedes aegypti* and *Ae. albopictus* has been demonstrated [28, 29]. A recent study has shown that *Wolbachia* can manipulate the mosquito cell RNAi/miRNA/piRNA machinery by inducing or suppressing specific small RNAs [30].

**Fig. 1:**
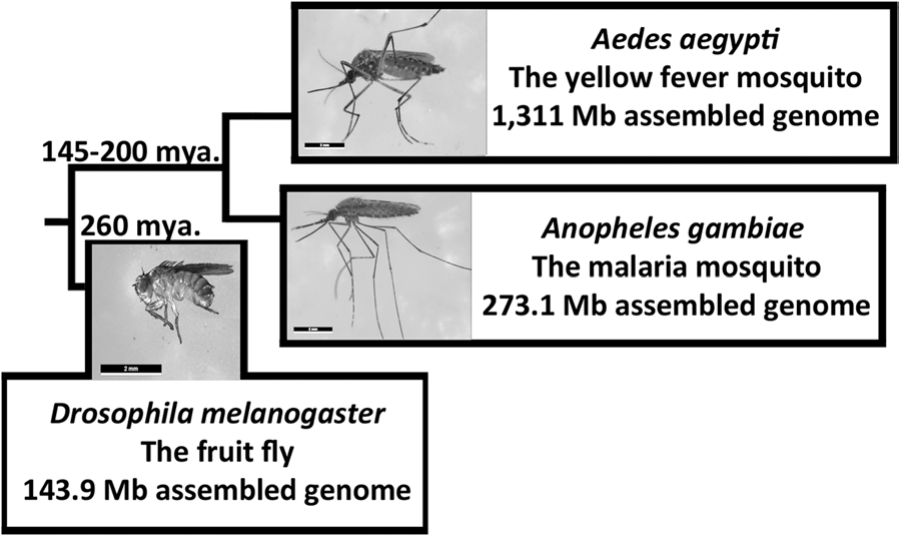
Phylogenetic delineation of three Dipteran species: *D. melanogaster*, *Ae. aegypti*, and *An. gambiae*

The majority of piRNAs originate from clusters, genomic regions ranging in size from approximately 1–250 kb [2, 22]. Produced piRNAs serve as guides for targeted inactivation of complementary TEs. piRNA clusters do not have an explicit strand bias; however, in some cases, they do exhibit high percentages of TEs in one orientation or the other [2, 31, 32]. In *D. melanogaster*, the piRNA clusters are almost exclusively located in heterochromatin—the pericentromeric and subtelomeric regions—regions with an abundance of TEs [2]. It is not clear if the predominant location of piRNA clusters in heterochromatin is specific to the fruit fly or is typical to Diptera. The African malaria mosquito, *Anopheles gambiae,* represents an intermediate in terms of the genome assembly size (273.1 Mb) [33] compared to other phylogenetically distant Dipterans with studied piRNA pathways *D. melanogaster* (143.9 Mb) [34] and *Ae. aegypti* (1311 Mb) [35] (Fig. 1). Moreover the genomic distribution of TEs differs among the three species. Over 77 % of pericentromeric heterochromatin and only 7 % of euchromatin are occupied by TEs in *D. melanogaster* [36]. This difference is less dramatic in *An. gambiae*: 33.1 % of pericentromeric heterochromatin and 14.5 % of the rest of the genome are covered by TEs [37]. The large regions of intercalary heterochromatin present in *An. gambiae* are mainly responsible for the high peaks of TE coverage outside the pericentromeric regions. In an extreme case, *Ae. aegypti* has a homogeneously high coverage of TEs (~52 %) across pericentromeric heterochromatin and other chromosomal regions [38]. Do mosquito species with a more ubiquitous distribution of TEs reflectively have a redistribution of piRNA clusters from heterochromatic to euchromatic regions? Does the piRNA pathway have conserved functions in organisms with different genome sizes, chromatin landscapes, and predominant TE families? These questions can be addressed by mapping piRNAs to annotated features of chromosome-based genome assemblies.

Here, we identified piRNAs from the ovarian tissue of *An. gambiae* females and characterized potential relationships between these small RNAs and the mosquito’s genomic features. We compared chromosome location of piRNA clusters in three Dipteran species with remarkably different patterns of genomic organization: *D. melanogaster*, *Ae. aegypti*, and *An. gambiae* [39]. We discovered a noticeable shift in piRNA cluster location in *An. gambiae* compared with *D. melanogaster*; the 15 most productive piRNA clusters are less confined to the pericentromeric heterochromatin, but can also be found in intercalary heterochromatin and euchromatin. The *Ae. aegypti* top 15 piRNA clusters [22] are even more pervasive, occupying euchromatic genomic scaffolds that have been previously placed to chromosomes by physical and genetic mapping [38, 40]. There is an increase in gene-derived piRNAs in the malaria mosquito when compared with the fruit fly. Among the genes identified in *An. gambiae* as rich in piRNA mapping, a subset includes genes potentially critical to reproduction and germline development, including *oskar* (AGAP003545).

## Results

### The piRNA population in the ovarian tissue of *An. gambiae*

**Fig. 2:**
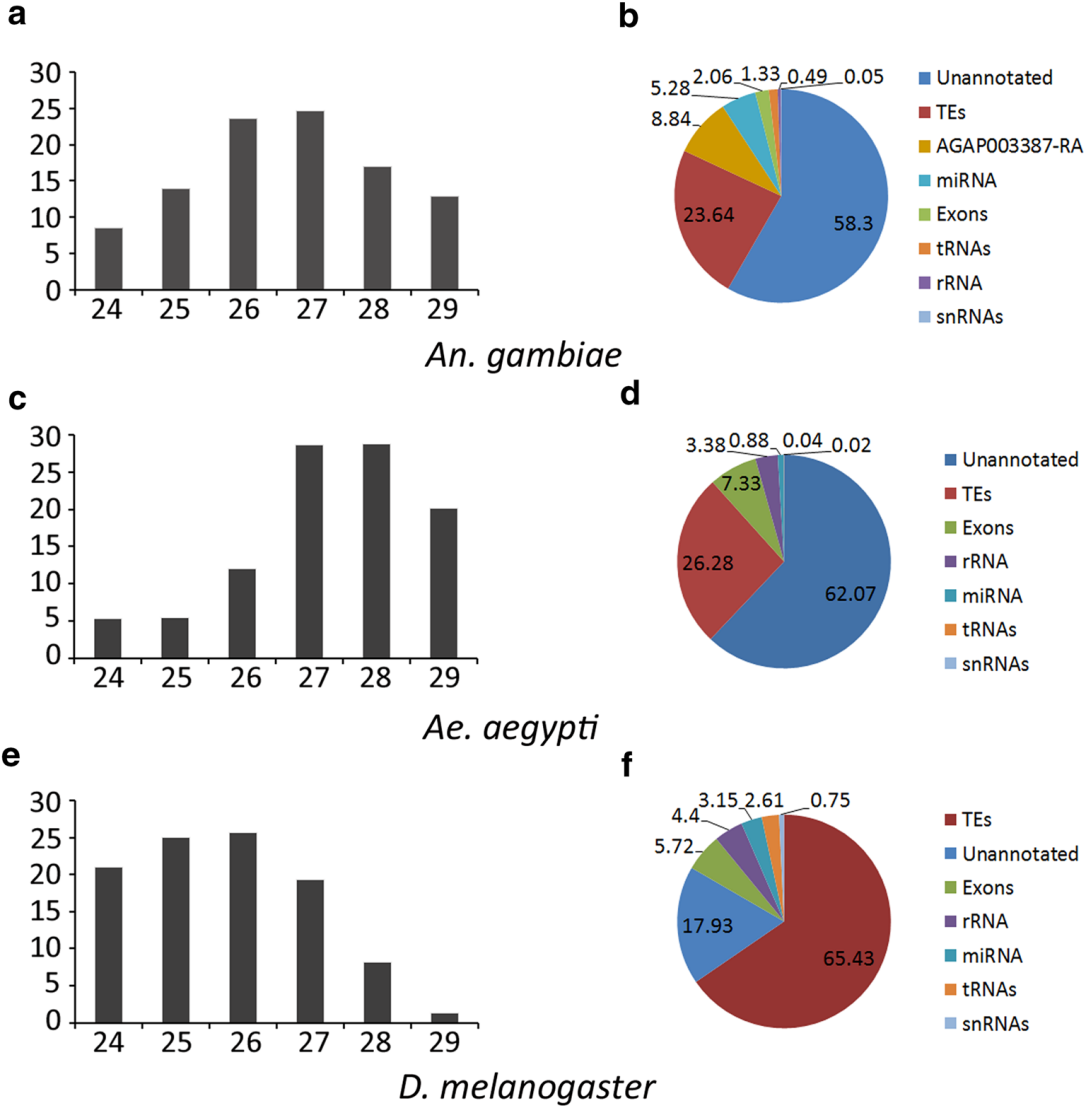
The size distribution and annotation of small RNAs within the piRNA size range (24–29 nt) of the NCNU libraries in Dipteran species *An. gambiae, Ae. aegypti,* and *D. melanogaster*. **a**, **c**, **e** represent the distribution of small RNA sequences found in the sequenced total library for the respective species (*X* axis: size in nucleotides (nt), *Y* axis: percentage of all 24–29 nt mapping small RNAs). **b**, **d**, **f** depict the annotation of these small RNAs for the three species

To accurately characterize the sequence and genomic location of piRNAs produced by the African malaria vector, we isolated and sequenced small RNAs from blood-fed ovaries of the Mali strain (the M form) of *An. gambiae* using the Illumina Small RNA TruSeq technology. The generated library of small RNAs, which we have named the non-collapsed non-unique (NCNU) library—reads including any duplicates (referred to as non-collapsed or NC) that map to one or more locations in the genome (referred to as non-unique or NU)—showed a bi-modal length distribution with two peaks (Additional file 1: Figure S1). The definition and sizes of each library are shown in Additional file 2: Table S1. The first peak occurred at a size of 22 nt, which we attributed to microRNAs (miRNA). The second broad peak spanning 24–29 nt with an apex at 27 nt represented the potential piRNA pool. The 27 nt peak has also been reported for the G3 strain (the S form) of *An. gambiae* [21, 23]. We noted that it differs from the 26 nt peak of *D. melanogaster* and 28 nt peak of *Ae. aegypti* (Fig. 2a, c, e). However, the total small RNA size range is in close concordance with *Ae. aegypti* (24–31 nt) [22], *D. melanogaster* (23–29 nt) [2], *Bombyx mori* (26–31 nt) [41], and *Danio rerio* (24–30 nt) [13].

### A majority of piRNAs target TEs (predominantly LTR retrotransposons) in both *An. gambiae* and *D. melanogaster*

To accurately characterize TE-derived piRNAs, 24–29 nt sequences from the NCNU library were mapped to the 7080 annotated Hexapoda TEs from RepBase [42]. The *An. gambiae* TE library is not nearly as extensive as the *D. melanogaster* TE library; therefore, we used all identified TE sequences from the subphylum Hexapoda to bolster the reference library used for piRNA mapping and to help mitigate potential non-annotated ancestral TEs. It is generally believed that the percentage of reported TE-matching piRNAs is an underestimation of the real percentage because of incomplete annotation of TEs in insect genomes [43]. Two approaches were taken to identify piRNAs derived from TEs: “consensus” mapping and “overlap” mapping. The first approach used traditional read mapping with Bowtie2, aligning the piRNAs to consensus TE sequences with up to three mismatches. This method relies on the assumption that three mismatches would be sufficient to identify TEs degraded from their original consensus sequences. Using this methodology, approximately 23.6 % of the total piRNA library was identified as being derived from TE sequences (Fig. 2b). We applied the same method to other Dipteran species with characterized piRNAs [22, 44] and found that this proportion of TE-mapped piRNAs is similar to that of *Ae. aegypti* (26.3 %) (Fig. 2d) but is much smaller than the 65.4 % identified in *D. melanogaster* (Fig. 2f). Some of this difference may be attributed to the larger proportion of unannotated sequences in *An. gambiae*, where at least 58.3 % of piRNAs are mapped to the unannotated part of the genome. This number is similar to that in *Ae. aegypti,* but is much larger than the 17.9 % identified in *D. melanogaster.*

The second approach of identifying TE-derived piRNAs sought to overcome limitations caused by a possible incomplete annotation of TE libraries and by severe sequence divergence from consensus TE sequences. Many of the TEs residing within a genome, especially within the heterochromatin, have been mutated to the point that they are quite different from their initial consensus sequences. By using up to three mismatches when mapping to consensus TE sequences, a substantial portion of low-identity TE sequences may be missed. We designed an “overlap” method that incorporated repeat masking data generated using the RepeatMasker platform [45] to identify genomic positions of the same 7080 annotated Hexapoda TEs sequences from RepBase [42]. Genomic piRNA positions were concurrently identified by short-read mapping the small RNAs to the organism’s reference genome assembly. The two position lists were overlapped to identify common genomic sequences that we considered TE-derived piRNA originators (see “Methods”). This second method resulted in 39.4 % of the piRNA pool that may be derived from TE sequences in the mapped portion of the *An. gambiae* genome (Additional file 3: Table S2). We tested the “overlap” approach for identifying TE-derived piRNAs on *D. melanogaster*. To do this, a *w*^*1118*^ ovarian piRNA library [44] was mapped to the *D. melanogaster* genome (version R6.04) with no mismatches. We used the same Hexapoda TEs from RepBase [42] to be consistent with our *An. gambiae* analysis. We found 81.6 % (2,149,899 of 2,634,680 RNAs) of the total sequenced piRNAs mapped to TEs in *D. melanogaster,* which is still much higher than the 39.4 % (8,890,061 of 22,569,568 RNAs) of TE-mapped piRNA identified in *An. gambiae* (Additional file 3: Table S2).

**Fig. 3:**
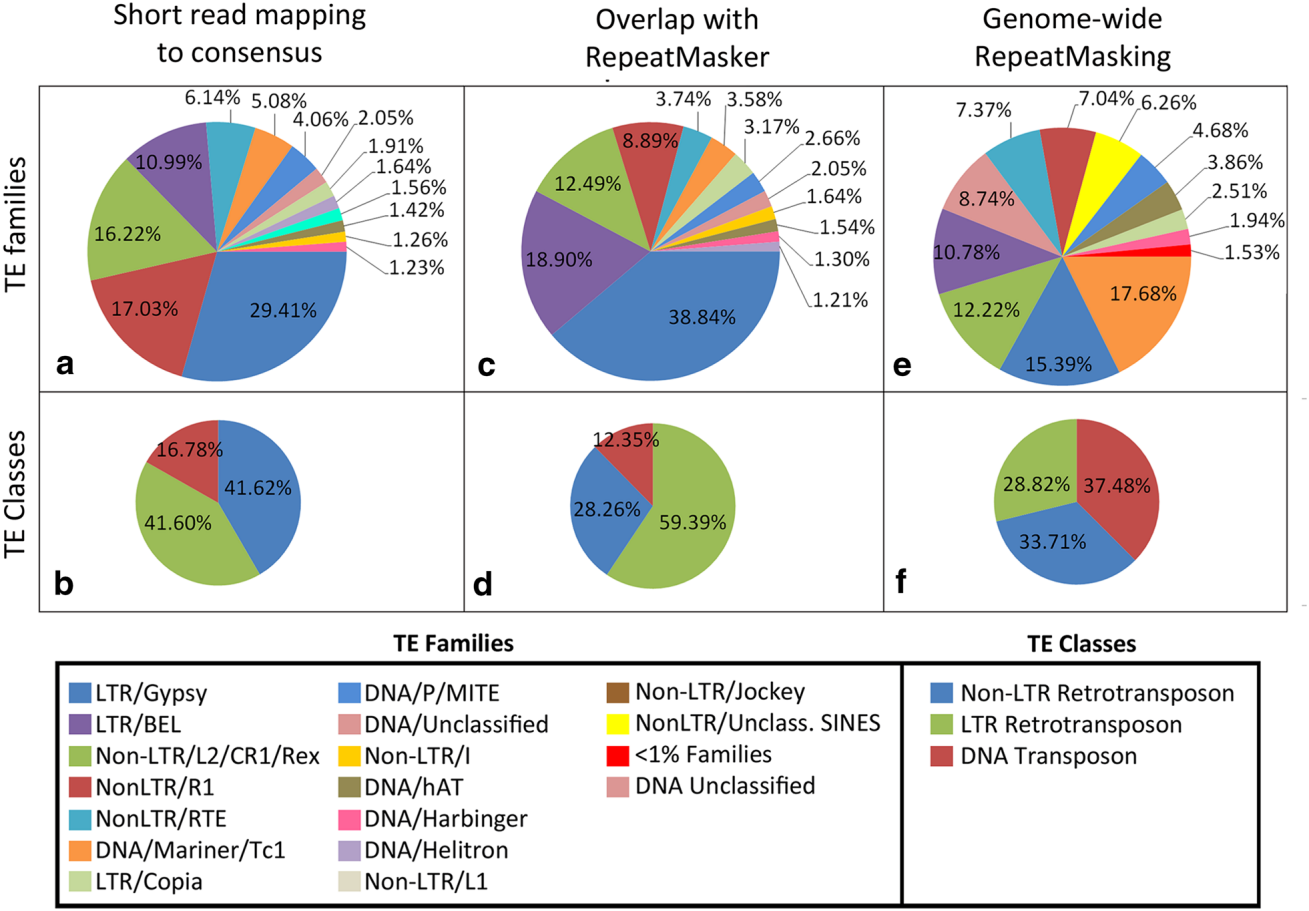
TE-derived piRNAs in *An. gambiae* identified by two different approaches. The percentage of TE-derived piRNAs mapping to the consensus sequences of annotated TEs with up to three mismatches in TE families (**a**) and TE classes (**b**). The percentage of TE-derived piRNAs with mapping positions that overlap RepeatMasker-identified positions in TE families (**c**) and TE classes (**d**). Genome-wide TE-content, comparison of TE families (**e**), and classes (**f**) by RepeatMasker. The Hexapoda library from RepBase [42] was used as reference

Although the two approaches for identifying TE-derived piRNAs result in considerably different predictions in respect to the percentage of piRNAs derived from TEs in *An. gambiae*, they still agree on the families of TEs with the highest abundance of mapped piRNAs (Fig. 3a–d). TE classes with the highest piRNA representation are LTR retrotransposons (59 % of piRNAs) followed by non-LTR retrotransposons (28 %) and DNA transposons (12 %), as determined by the “overlap” method. However, classes and families most abundant in piRNA mapping do not correspond to the TE classes and families most prevalent in the *An. gambiae* genome (Fig. 3e, f). For example, LTR retrotransposons represent the most abundant class of TEs enriched in piRNAs (Fig. 3b, d). Yet, non-LTR retrotransposons are more abundant in the *An. gambiae* genome than are LTR retrotransposons (34 vs. 29 %, respectively, Fig. 3f). Moreover, 37 % of all TE sequences in the *An. gambiae* genome are represented by DNA transposons, while DNA transposons generate only 12 % of piRNAs (Fig. 3d).

### TE mapping data provide evidence of an active ping-pong amplification cycle in *An. gambiae* ovaries

In the ping-pong amplification cycle, antisense piRNAs are considered to be almost exclusively derived from piRNA clusters and are proposed to be primarily responsible for TE silencing through the piRNA pathway [2]. In the *An. gambiae* dataset, we identify a strong bias toward antisense piRNAs. About 69 % of the piRNAs identified as TE-derived were found in the opposite orientation with regard to TEs identified by the “overlap” method. This finding holds consistent across each of the arms and to an extent within the unknown (UNKN) chromosome, which consists of unmapped genomic scaffolds (Additional file 4: Figure S2).

In addition, the ping-pong amplification loop promotes the generation of both sense and antisense RNAs that help to guide piRNA complexes toward transposon mRNAs for their subsequent processing. The RNAs generated from this amplification loop typically exhibit two motifs. Both motifs, a precise 10 nucleotide overlap between overlapping piRNAs and positional biases with Uridine at position 1 and Adenine at position 10 [2, 8, 22], stem from the way in which the ping-pong amplification generates piRNAs. In *D. melanogaster*, Piwi and Aubergine proteins have a noted preference toward piRNAs that are antisense to mRNAs encoded by TEs with a Uridine at the 5′ end [2]. As the PIWI-mediated cleavage is now known to occur between positions 10 and 11 of the RNA complementary to the guide strand, the resulting secondary, sense piRNA is expected to contain an Adenine at the tenth nucleotide. Ago3 proteins have a preference toward piRNAs sense to TE mRNAs. This targeted cleavage results in an amplified piRNA pool capable of targeting both sense and antisense transposon transcripts.

**Fig. 4:**
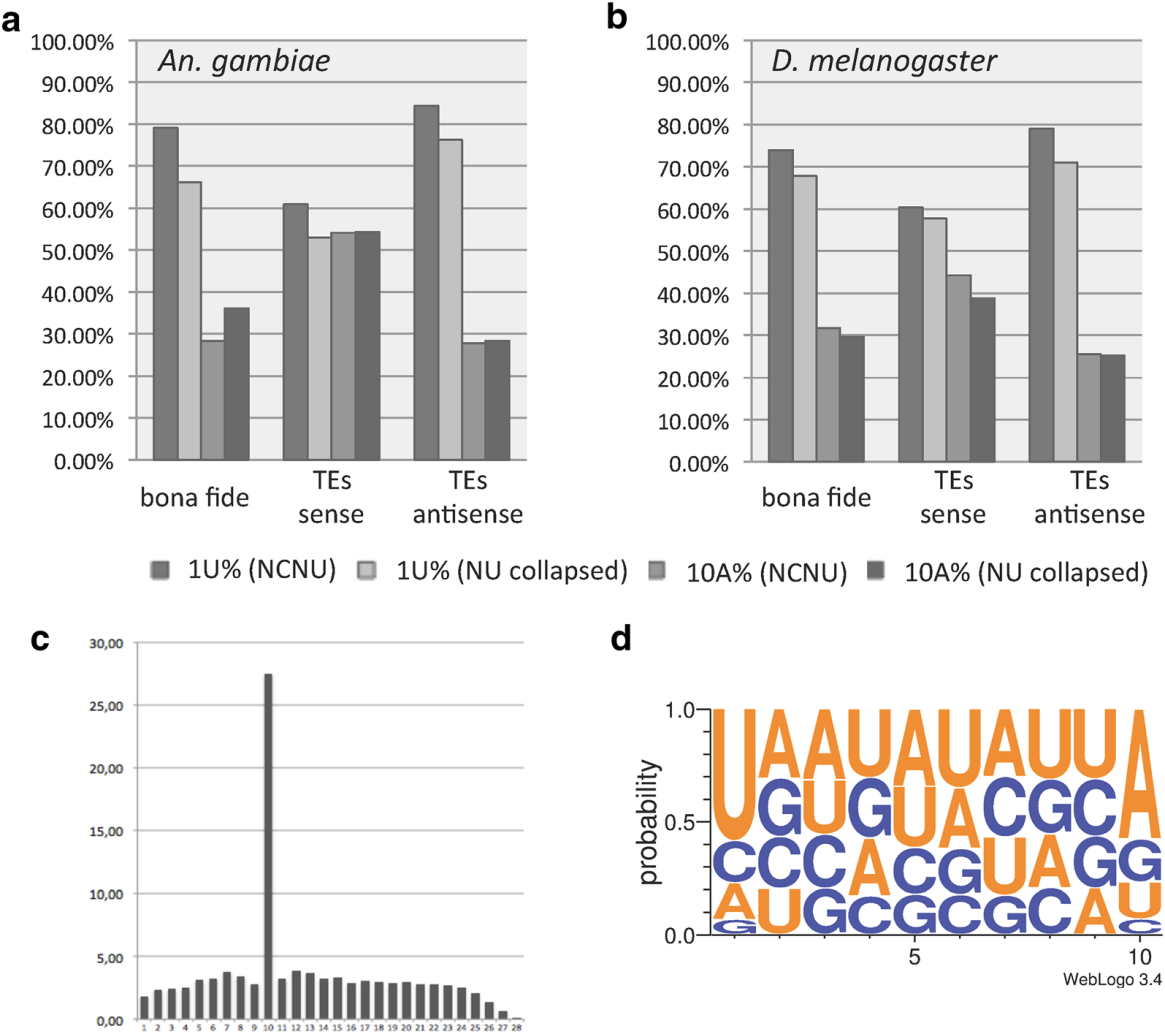
Characteristics of piRNA sequences (24–29 nt). **a** Percentages of 1U and 10A signatures in bona fide and in TE piRNAs in *An. gambiae.* **b** Percentages of 1U and 10A signatures in bona fide and in TE piRNAs in *D. melanogaster*. **c** 5′ ends of complementary piRNAs of *An. gambiae* frequently exhibited a 10-bp overlap (the ping-pong signature) in all piRNA genome-mappers. **d** Nucleotide abundance in the 10-bp overlap section of ping-pong piRNA partners in *An. gambiae*

From a total of 22,569,568 small RNAs ranging from 24 to 29 nt and constituting the potential piRNA pool in *An. gambiae*, we further examined this population for piRNA signatures and for sequences from which these piRNAs might be derived. We identified 79.1 % of the 24–29 nt bona fide reads (excluding miRNA, rRNA, tRNA, snRNA) as having a U at position 1 and 28.4 % as having an A at position 10 (Fig. 4a). We also analyzed TE-mapping piRNAs separately, allowing 0–3 mismatches. Using the NCNU library, the sense reads for TEs show a 60.9 % bias of 1U and a 54.0 % bias of 10A, while antisense reads show a 84.3 % bias of 1U and a 27.8 % bias of 10A. The values obtained from the collapsed NU library are very similar (Fig. 4a), indicating that 1U and 10A biases are not due to multiplicity of many piRNA reads. These percentages are very close to the corresponding percentages in *D. melanogaster* (Fig. 4b). We detected a strong 10 nt overlap bias (Fig. 4c) with 1U and 10A signatures (Fig. 4d) in the 24–29 nt subgroup from *An. gambiae*. In accordance with previous studies [21, 23], the presence of these signatures suggests that a mechanism similar to the established ping-pong amplification loop in *D. melanogaster* exists in the ovarian tissue of *An. gambiae*, functioning with Ago3 and Aub orthologs, which load preferentially sense and antisense piRNAs, respectively.

## 187 piRNA clusters are identified in the genome of *An. gambiae*

The *An. gambiae* PEST genome is chromosomally assembled [33, 46], allowing for the spatial identification of chromosomal positions associated with piRNA enrichment. The reference PEST strain has haplotypes of both S and M forms of *An. gambiae* segregating in different regions of the genome [33]. Overall, the M form genome is slightly more similar to the PEST genome than is the S form genome [47], which gave us an advantage in mapping the Mali strain (the M form) reads to the reference genome. piRNA clusters in *An. gambiae* were identified using a strategy that incorporated the methods used previously in both *D. melanogaster* [2] and *Ae. aegypti* [22] (see “Methods”). A total of 120 piRNA clusters in *An. gambiae* were found on the assembled chromosomes, with 18 clusters being identified on chromosome X, 35 clusters on 2R, 27 on 2L, 18 on 3R, and 22 on 3L (Additional file 5: Table S3). Chromosome UNKN, being made up of variously sized scaffolds sorted in decreasing order by length, provides approximately 40 % of the mapped piRNA sequences. The abundance of small, artificially concatenated scaffolds in the UNKN chromosome made it difficult to determine the boundaries of piRNA clusters when working with 5-kb windows, so we limited our analyses to the first 10 Mb. Scaffolds at the 10-Mb region of chromosome UNKN were at least ~50 kb in length. We identified 67 extra clusters present in the first 10 Mb of chromosome UNKN. The total 187 clusters span in size from 10 to 1.29 Mb, which is a departure from the smaller clusters (2–242 kb, 6–184 kb, respectively) identified in previous studies in *D. melanogaster* [2] and *Ae. aegypti* [22]. We ran our piRNA cluster analysis pipeline using the *w*^*1118*^ piRNA library [44] on the Dmel_R6.04 release of the *D. melanogaster* [34] genome assembly using the same methodology as for *An. gambiae* in an effort to compare genomic location of piRNA clusters in the different species (see "Methods"). We identified 155 clusters in *D. melanogaster* ranging in size from 10 to 1.13 Mb (Additional file 5: Table S3).

### A large proportion of *An. gambiae* piRNA clusters is uni-directionally transcribed

**Fig. 5:**
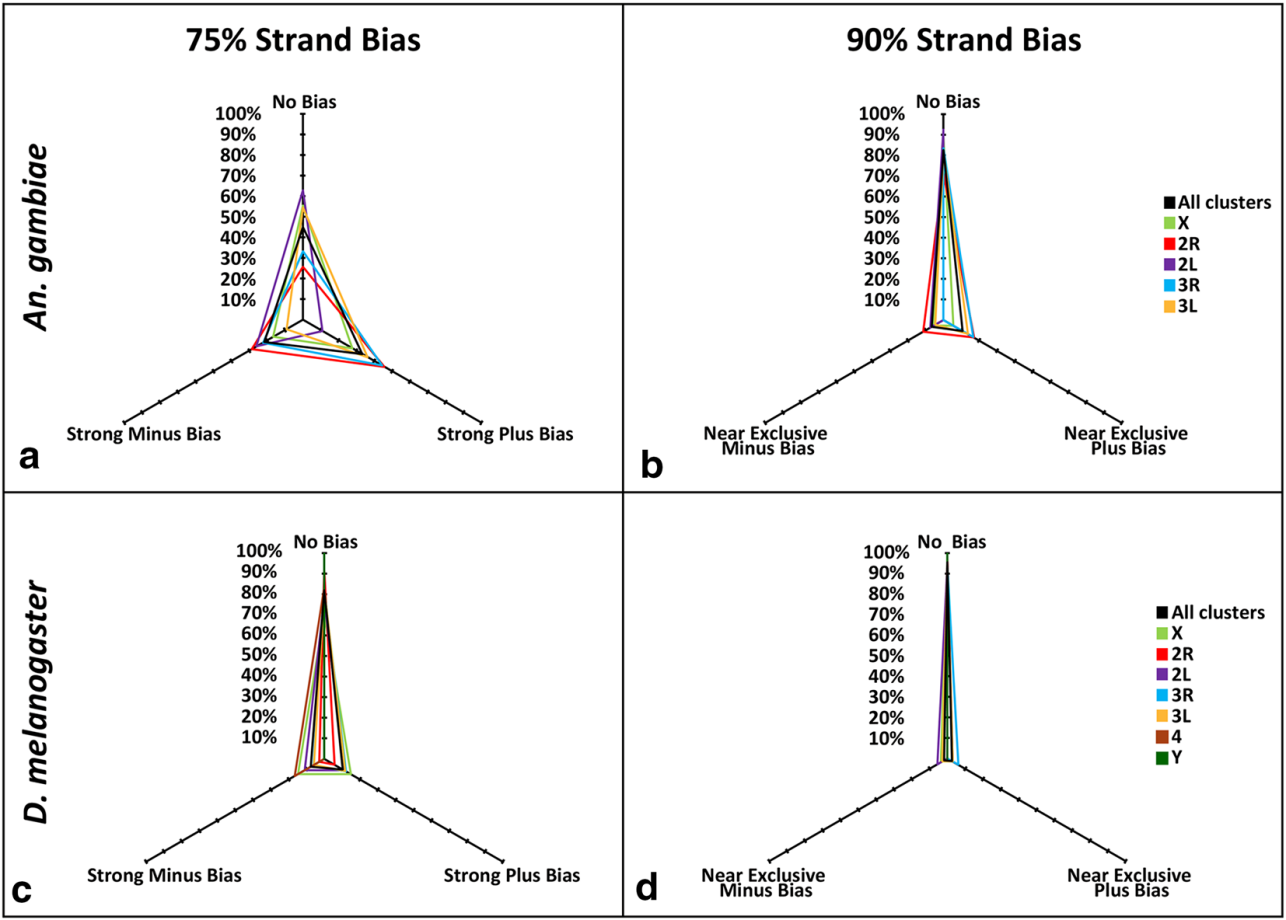
*Anopheles gambiae* has more unidirectional piRNA clusters than does *D. melanogaster*. Spider chart depicting bias (>75 % of piRNAs map to one genomic strand) (**a**, **c**) or (>90 % of piRNAs map to one genomic strand) (b, d) for clusters per chromosomal arm as well as all clusters combined. The percentage of clusters on each arm (or total) is displayed for either bias or no bias in *An. gambiae* (**a**, **b**) and *D. melanogaster* (**c**, **d**)

A majority of ovarian piRNA clusters are bi-directionally transcribed in *D. melanogaster* [2]. Unidirectional clusters, like *flamenco*, are primarily expressed in the somatic follicular cells, while bidirectional clusters are transcribed in ovarian nurse cells [48]. In *An. gambiae*, 66 of the 120 clusters (55 %) belonging to assembled chromosomes had more than 75 % of piRNAs mapping to a single strand (strong bias) (Fig. 5a). Still, 17.5 % of mapped piRNA clusters had more than 90 % of piRNAs mapping to only a plus or minus strand (near exclusive bias) (Fig. 5b). In contrast, only 18 % of piRNA clusters had a strong plus or minus strand bias in *D. melanogaster* (Fig. 5c). Furthermore, only 4.5 % of piRNA clusters had a near exclusive bias of piRNAs mapping to a single strand in *D. melanogaster* (Fig. 5d). If we consider all 187 piRNA clusters in *An. gambiae*, 38 % had a strong strand bias and 11.2 % had a near exclusive strand bias. This result indicates that the UNKN chromosome clusters, which are likely heterochromatic, are mostly bidirectional. The very top piRNA cluster, which is located in the *An. gambiae* euchromatin, is unidirectional (Additional file 6: Figure S3A). The next two top piRNA clusters are bidirectional and are located in intercalary and pericentromeric heterochromatin of *An. gambiae* (Additional file 6: Figure S3B, C).

### Genomic location of piRNA clusters vary among three dipteran species

**Fig. 6:**
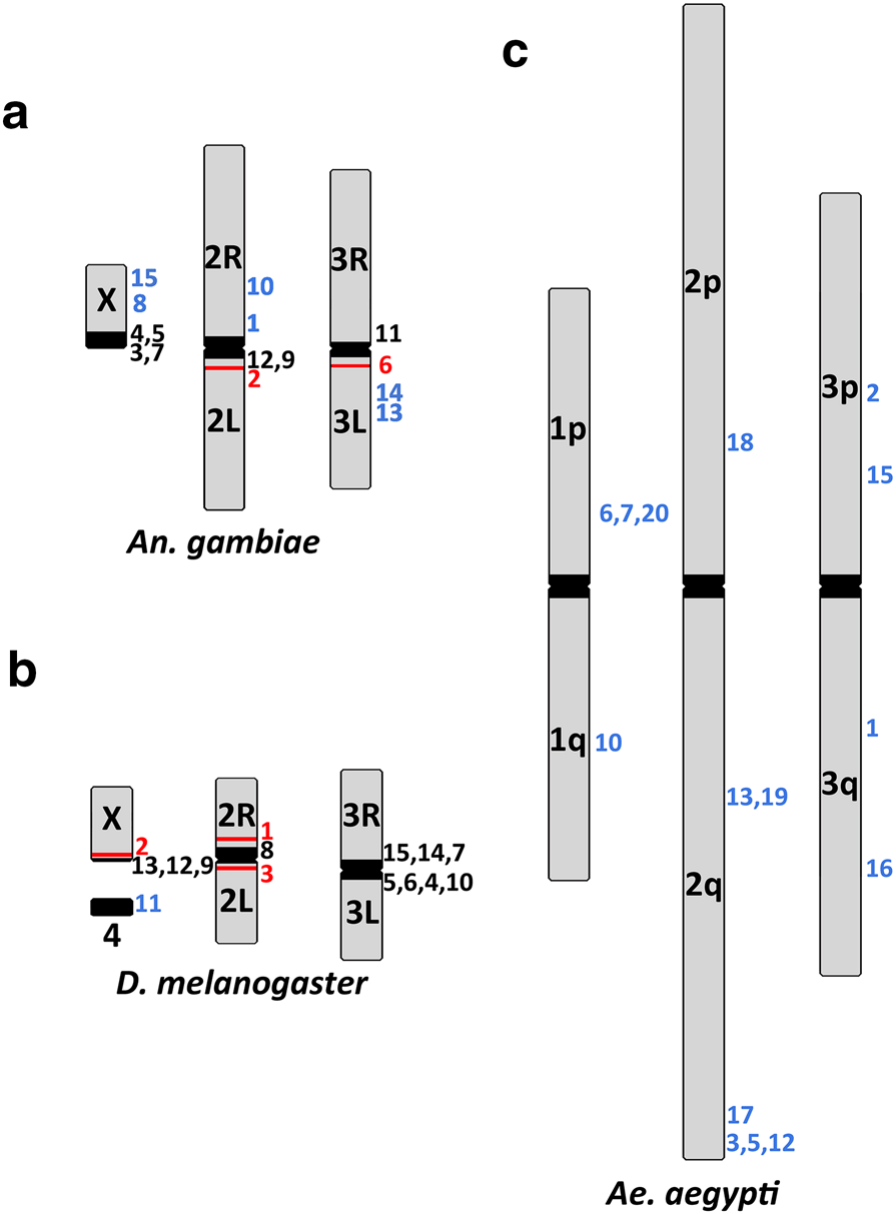
The chromosome distribution of the top 15 mappable piRNA clusters within three Dipteran species. **a** *An. gambiae*, **b** *D. melanogaster*, **c** *Ae. aegypti*. Clusters are numbered to the right of the chromosome arms, with *colors* (*gray* euchromatic, *black* pericentromeric heterochromatin, *red* intercalary heterochromatin) indicating predicted chromatin type

Of the total 120 chromosomally mapped piRNA clusters in *An. gambiae*, the longest piRNA clusters localized to heterochromatic regions [37]. Twenty-six of the clusters (21.7 %) were located in pericentromeric heterochromatin; two were found in the intercalary heterochromatin of arms 2L and 3L, and 92 piRNA clusters occupied euchromatic regions of all chromosomes (Additional file 5: Table S3). Of the top 15 piRNA clusters (ranked by the number of unique piRNAs), seven (46.7 %) were located in pericentromeric heterochromatin of chromosomes X, 2L, and 3R, two clusters were located in intercalary heterochromatin of arms 2L and 3L, and six piRNA clusters occupied euchromatic regions of chromosomes X, 2R, and 3L (Fig. 6a).

Of the total 155 piRNA clusters identified in *D. melanogaster*, 76 (49 %) were located in pericentromeric heterochromatin. In addition, 26 clusters were found in intercalary heterochromatin [49], and 2 clusters were mapped to Y-chromosome heterochromatin. The remaining 51 piRNA clusters occupied euchromatic regions. All but three of the top 15 clusters identified by Brennecke et al. [2] correspond to the clusters with the highest number of unique piRNAs identified using our methodology. Unlike the previous study [2], we classified the three highest piRNA-producing clusters in subdivisions 2R:42AB, X:20A, and 2L:38C as intercalary heterochromatin instead of pericentromeric heterochromatin based on the presence of late replication sites and location of Suppressor of Under Replication (SuUR) in wild-type flies [49]. The cluster in region X:20A included the *flamenco* locus and the second top cluster from an earlier study [2]. Our approach was designed to determine genomic loci that are responsible for the most abundant production of piRNAs in order to compare piRNA clusters between species, rather than to identify precise boundaries of clusters. We found that 11 of the top 15 chromosomally mapped piRNA clusters are pericentromeric, while the remaining clusters were within intercalary heterochromatin or the euchromatin of chromosome 4 (Fig. 6b).

To determine the chromosomal location of the top piRNA clusters in *Ae. aegypti* [22], we identified the cluster containing supercontigs on the physical [38] and linkage [40] maps of this species. We were able to find chromosomal locations for 24 of top 30 piRNA clusters of *Ae. aegypti* reported previously [22] (Additional file 7: Table S5 and Additional file 8: Figure S6). None of these clusters were located in pericentromeric heterochromatin, but all major clusters were detected in euchromatic regions of all three chromosomes of *Ae. aegypti* (Fig. 6c). Analysis of these data provide evidence that the degree of confinement of the top 15 piRNA clusters to the pericentromeric heterochromatin varies among the three dipteran species from 73.3 % in *D. melanogaster* to 46.7 % in *An. gambiae* to 0 % in *Ae. aegypti*. If we consider piRNA clusters from the UNKN chromosome of *An. gambiae*, four of them would be among the top 15 clusters (ranking 4, 12, 13, 14). However, we cannot assign them to any heterochromatic or euchromatic region.

### piRNA production shifted from pericentromeric regions to the rest of the genome in the mosquito compared with the fruit fly

We investigated possible differences in piRNA production between *An. gambiae* and *D. melanogaster* when considering the genomic location of piRNA clusters. A previous study indicated that pericentromeric clusters are the primary production sites of piRNAs in *D. melanogaster* [2]. Although we reclassified the three highest piRNA mapping pericentromeric clusters as intercalary heterochromatin, we still found that pericentromeric regions in *D. melanogaster* produce 41.6 % of all genome-unique piRNAs (Additional file 9: Table S4). In contrast, pericentromeric clusters in *An. gambiae* produce only 24.8 % of unique piRNAs, excluding the UNKN chromosome (Additional file 5: Table S3). Even if we include the UNKN chromosome in the analysis and artificially assign all UNKN chromosome clusters to the pericentromeric heterochromatin, still only 34.3 % of unique piRNAs would be produced by the pericentromeric clusters in *An. gambiae.* In addition, 29.3 and 6.6 % of unique piRNAs are derived from euchromatic clusters in *An. gambiae* and *D. melanogaster,* respectively, if we exclude the UNKN chromosome. Still, euchromatic clusters in *An. gambiae* would produce 17.6 % of unique piRNAs, if we include the UNKN chromosome in the analysis. Another interesting difference we found is that as much as 43.2 % of total unique piRNAs is produced outside clusters identified in our study in *An. gambiae* vs. 22 % in *D. melanogaster,* further supporting the shift in piRNA production from pericentromeric regions to the rest of the genome when comparing the mosquito with the fruit fly.

To delve into possible reasons for the difference in piRNA production, we compared TE distribution landscapes between the mosquito and fruit fly. We determined the TE content in the genomes and piRNA clusters using RepeatMasker [45] and a library of 7080 annotated Hexapoda TEs sequences from RepBase [42]. Only 41.8 % of pericentromeric heterochromatin was covered by TEs in *An. gambiae* (Additional file 10: Figure S4), while 77.3 % of pericentromeric heterochromatin was occupied by TEs in *D. melanogaster* (Additional file 11: Figure S5). The rest of the genomes had a similar TE coverage: 6.7 % in *An. gambiae* and 5.5 % in *D. melanogaster.* There was good correspondence between piRNAs and TE sequences in the *An. gambiae* (Additional file 10: Figure S4) and *D. melanogaster* (Additional file 11: Figure S5) genomes. Of the 120 total mapped piRNA clusters in *An. gambiae*, the 26 pericentromeric clusters were enriched in TE sequences (48.1 %) in comparison with the remaining heterochromatic and euchromatic clusters (22.8 % TE content) (Additional file 5: Table S3). Altogether, the 120 mapped clusters had an average TE content of 31 % (Additional file 5: Table S3). The clusters identified on chromosome UNKN had ~40 % TE content, suggesting that they are likely unassembled sequences belonging to the pericentromeric and/or intercalary heterochromatin. However, a much more dramatic difference in TE content could be seen in *D. melanogaster* between the 76 pericentromeric clusters, having an average of  84.3 % TE content and the remaining heterochromatic and euchromatic piRNA clusters having 32.0 % TE content (Additional file 9: Table S4). The very high abundance of TEs in *Drosophila* pericentromeric heterochromatin could be responsible for increased piRNA production by this genomic domain of the fruit fly. On the other hand, the top 24 mapped clusters in *Ae. aegypti*, which are mainly euchromatic (Additional file 8: Figure S6), have a mean TE content of 47 % (Additional file 7: Table S5) [22]. Thus, the lower overall TE content in piRNA clusters of *An. gambiae* compared to that in two other species suggests that other sequences (unidentified TEs or genes) could serve as progenitors of piRNAs.

We documented a marked shift in major piRNA cluster location: from mainly pericentromeric heterochromatin in *D. melanogaster*, to both intercalary heterochromatin and euchromatin in *An. gambiae*, to mainly scattered euchromatic regions in *Ae. aegypti* (Fig. 6). The intercalary heterochromatin regions in *An. gambiae* are, like the pericentromeric heterochromatin, high in TE content and low in genic content [37]. Two regions of diffuse intercalary heterochromatin regions are responsible for a substantial portion of the piRNA population. However, one region of compact intercalary heterochromatin, located in subdivision 35B of 3R arm, does not have enough piRNA enrichment to be considered a cluster. Compact intercalary heterochromatin has a different composition than the diffuse intercalary and pericentromeric heterochromatin. For example, the Ty3/gypsy TEs represent 6.9 % of the diffuse intercalary heterochromatin and only 1.6 % of the compact intercalary heterochromatin in *An. gambiae*. piRNAs mapped to Ty3/gypsy represent the most substantial class of TE-mapped piRNAs. In addition, subdivision 35B of 3R arm has a higher enrichment of solo-LTR retrotransposons than any other region in the genome (euchromatic or heterochromatic) [50].

### *An. gambiae* ovaries have an abundance of gene-derived piRNAs and siRNAs

In *An. gambiae*, a peak of 21-nt siRNAs is observed for gene transcript mapping small RNAs (Additional file 12: Figure S7). One of the most notable roles for siRNAs is within the RNA interference (RNAi) pathway, where the endogenous siRNAs help to regulate gene expression through Dicer-mediated cleavage [8]. Exogenous siRNAs, on the other hand, play crucial roles in the defense against RNA arboviruses in *Aedes* mosquitoes [28, 29]. However, the exogenous siRNA pathway plays no detectable role in antiviral defense in the midgut of *An. gambiae* [51]. We show that gene-derived small RNAs from ovarian tissue comprise both siRNA (21 nt) and piRNA (24–29 nt) populations in different proportions, depending on the genes (Additional file 12: Figure S7). Some transcripts have relatively high levels of siRNA mapping (Additional file 12: Figure S7B, C). As piRNAs are more widely associated with TE sequences, the presence of a large fraction of gene-derived piRNAs, one that is larger than even the siRNA contingent (Additional file 12: Figure S7A), is an interesting observation. By looking at their mapping sites within transcripts, we further analyzed this sub-population of genic piRNAs, as well as the piRNA-enriched gene’s functions.

**Fig. 7:**
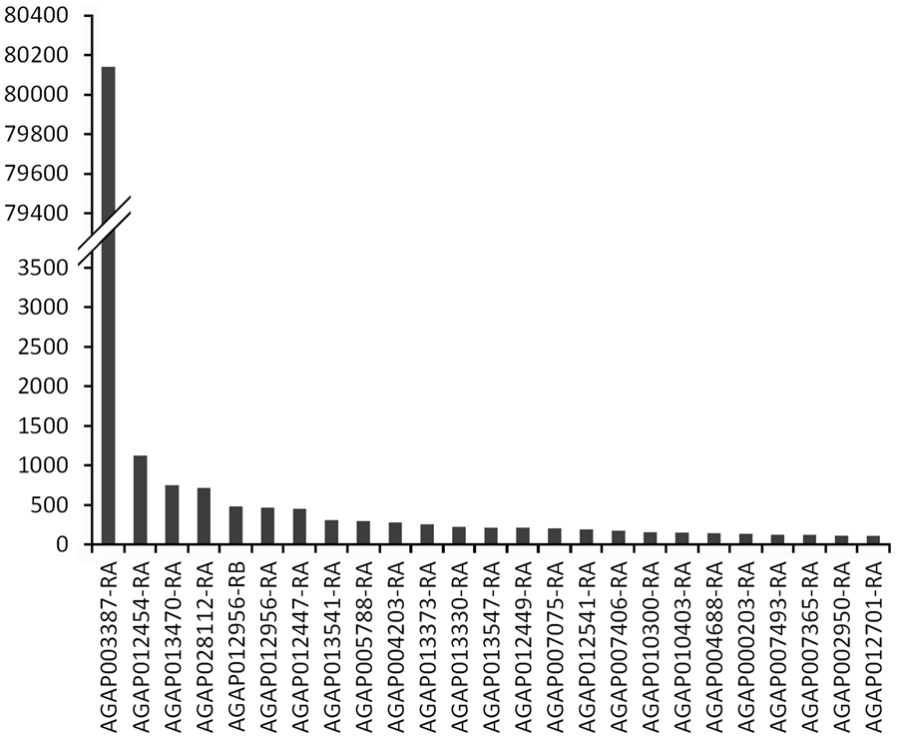
The *An. gambiae* genes with substantial piRNA enrichment. The top 25 genes with piRNA mapping are shown. The mapping includes AGAP003387, accountable for 8 % of genic piRNAs. The *Y* axis indicates the number of reads per million genome-mappers

Previous reports have shown that a majority of piRNAs associated with genes are derived from the 3′ UTRs of the transcripts [19–23]. The 3′ UTR has been associated with mRNA localization, translation, and stabilization [52], which all support post-transcriptional regulation of gene expression. MicroRNAs (miRNAs), another class of small RNAs, can bind to the 3′ UTR region of transcripts and affect gene expression through translational inhibition or transcript degradation [53]. To evaluate if piRNAs play a role in regulation of gene expression in *An. gambiae*, the original small RNA library was modified to contain non-collapsed, unique (NCU) piRNAs totaling 6,805,309 sequences ranging from 24 to 29 nt (Additional file 2: Table S1). We localized the NCU piRNA library to annotated *An. gambiae* gene transcripts and identified 5024 genes with more than 0.5 reads per million (RPM) genome-unique piRNAs that mapped within the transcripts. piRNA reads were normalized using RPM in order to compare mapping trends between *An. gambiae* and *D. melanogaster*. However, upon further analysis, many gene transcripts appear to have TE sequences within them. We identified 69 transcripts from 65 genes that gave more than 0.5 RPM of TE-matching piRNAs (allowing 0–3 mismatches) (Additional file 13: Table S6). Five of these genes, AGAP012494-RA, AGAP003870-RA, AGAP001582-RA, AGAP005927-RA, and AGAP000983-RA, had more than half of the piRNA reads mapped to TEs. We analyzed in detail the 65 genes and their piRNAs. The gene sequences that map to TE-matching piRNAs are mostly restricted to short stretches of 30–100 nt. There is one exception with AGAP012494-RA, where TE-matching piRNAs cover 660 nt, corresponding to 90 % of the transcript. The AGAP012494-RA transcript has 90 % sequence identity over 685 nt to AgaP8MITE2450, a DNA transposon. The gene generates 183.8 RPM piRNAs, of which, 162.4 RPM piRNAs map to AgaP8MITE2450 when allowing 0–3 mismatches. These data suggest that AGAP012494 is derived from an ancient AgaP8MITE2450 element. All transcripts’ piRNAs, which also map to TEs when allowing 0–3 mismatches, equal 14,350 reads (859.7 RPM). About half of these piRNAs, 49.6 %, map to RepBase reference TEs from *Anopheles* without any mismatches, suggesting a rather recent origin of the corresponding sequences within protein-coding gene transcripts. The piRNAs map to many types of TEs: SINEs, LINEs, Gypsy-like, BEL-like and DNA transposons (Additional file 14: Table S7). Still, a large number of these piRNAs, 5346 reads, map to SINEX-1_AG, a SINE element. We then removed the piRNAs that map to both gene transcripts and TEs, and used the remaining set of piRNAs for further transcript analysis. We identified a single euchromatic gene, AGAP003387, which had 88,391 RPM bona fide piRNAs (24–29 nt) (81 % of the genic piRNAs) localized to its transcript’s sequence (Fig. 7). Nearly all of these hits correspond to two short regions within the 3′ UTR of AGAP003387, in agreement with previous studies [21, 23]. AGAP003387 is a putative lipoprotein gene with orthologs restricted to the closely related species of the *An. gambiae* complex. This gene has no significant resemblance to any currently identified TEs, and shows a similar RNA expression profile to a variety of protein-coding genes. Expression of AGAP003387 significantly increases 9.3-fold between 0 and 10-day-old adult female mosquitoes [54]. Exclusion of AGAP003387 from the analysis results in 2.1 % of the total piRNA pool being generated from transcripts of the remaining gene dataset (Fig. 2b). Nearly 58 % of the unique piRNAs that mapped to genes were located within the 3′ UTR of gene transcripts. Approximately 800 genes with any piRNA mapping had piRNA reads entirely derived from the 3′ UTR.

piRNAs have been mapped primarily to the sense strand of various genes in *D. melanogaster* [19, 20] and *M. musculus* [55], including *Traffic jam*, *brat*, and *Klp10.* We saw a similar phenomenon in *An. gambiae*, where 81 % of genome-unique piRNAs matching protein-coding transcripts were derived from the sense strand. This, in concordance with previous reports [21–23], likely suggests that in mosquitoes, there is a mechanism that generates piRNAs from various non-TE-related sequences, protein-coding genes in particular. Most of these piRNAs, lacking their complementary antisense partners, are not fed through the ping-pong amplification cycle. The resulting sense bias of these piRNAs indicates that the small RNAs do not serve as complementary guides for targeted transcript degradation as characterized in TE regulation. Rather, it is more likely that these piRNAs interact directly with sequences elsewhere in the genome, or have a yet unknown role in gene regulation.

### piRNA-producing genes play a role in regulation of gene expression, reproduction, and development in *An. gambiae*

The piRNAs stemming from non-TE sources are less studied, and the process and function of these piRNAs have yet to be elucidated. As part of this study, we aimed to identify genes enriched in piRNA mapping and to classify them based on predicted Gene Ontologies (GO). Functional annotation of these genes may provide insight as to why some transcripts are at the origin of a large subset of piRNAs and when these piRNAs may be generated. Transcripts for annotated genes were used as reference sequences to identify piRNAs derived from RNA sequences post-splicing. Based on prior piRNA mapping enrichment, genes were assigned to three groups for further exploration: genes with a unique-mapping piRNA RPM of 5–10, genes with an RPM of 10–50, and genes with an RPM higher than 50. Each list of genes was submitted to the DAVID functional annotation tool [56, 57] to determine potential GO terms.

The DAVID annotation tool provides automatic clustering capabilities, resulting in collections of genes with similar functions and processes. We used an EASE score cutoff of greater than 1.3 to recognize relevant annotation clusters. The EASE score represents the mean of *p* values from a cluster, where a score of 1.3 is equivalent to a *p* value of 0.05. The 5-10 RPM piRNA group consisted of 342 annotated genes that were separated into 14 significant clusters. The 10–50 RPM piRNA group contained 263 annotated genes, creating six distinct, significant annotation clusters (Additional file 15: Table S8). Forty-six annotated genes had more than a 50 RPM, with no significant biological meanings being shared amongst the group. GO term analysis resulted in many various predicted functional annotation terms (FATs) for the two gene subsets with significant clustering. From the different FATs that were identified, similar broad functions for the most enriched clusters of both subsets could be identified. FATs for the 5–10 RPM piRNA group range from translation initiation, protein binding, DNA and RNA binding, methyltransferase activity, to chromatin binding. Many of these FATs suggest potential roles in regulation of gene expression and chromosome organization.

**Fig. 8:**
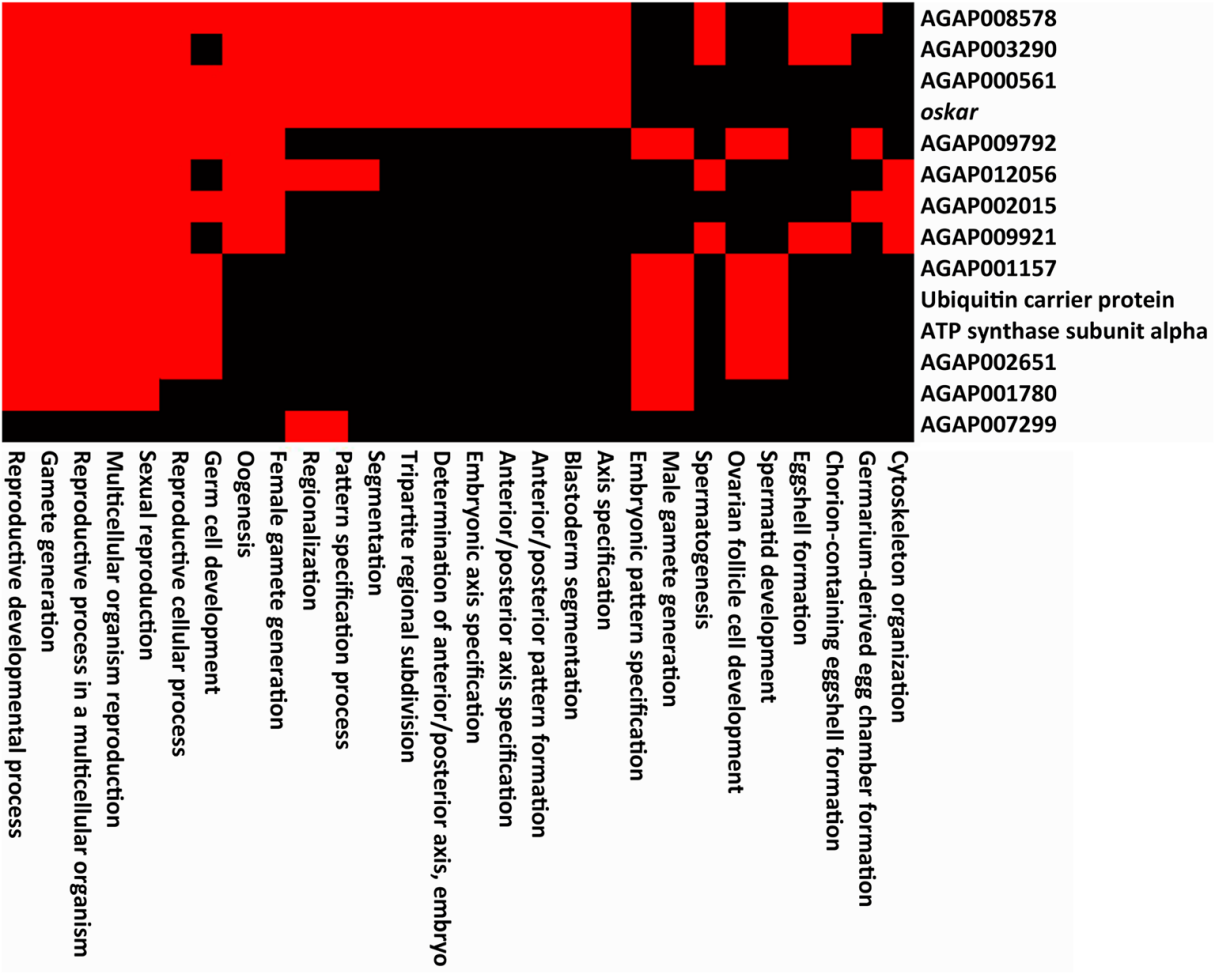
DAVID functional annotation terms of the “reproduction and development” cluster genes with 10–50 RPM mapped unique piRNAs in *An. gambiae*. Genes within the cluster (*Y* axis) are overrepresented by GO terms (*X* axis), with many of the genes sharing similar predicted functions (*red boxes*)

FATs for the 10–50 RPM piRNA group included chromatin assembly and organization, nucleosome assembly and organization, protein transport and localization, and regulation of translation. These terms suggest that proteins from this subset of genes are also involved in regulation of gene expression. One cluster of 14 genes, comprising the third most enriched cluster of the 10–50 RPM piRNA group, was identified to represent functions related to development, gamete generation, and sexual reproduction (Fig. 8). Four of these genes are found within piRNA clusters (AGAP000145, AGAP001157, AGAP002651, AGAP005134), and the rest are located outside. Because reproduction and development of mosquitoes are important biological functions to target while designing novel vector control strategies, the identification of a number of piRNA-enriched genes associated with these functions warrants further investigation. For example, *oskar* (AGAP003545), one of the 14 “reproduction and development” cluster genes, produces mRNAs that are restricted to female ovaries and the posterior pole of mosquito embryos [58]. In *D. melanogaster,oskar* is associated with pole plasm determination and axis specification within oocytes [59]. The *Drosophilaoskar* has been shown to be repressed in early oocyte development, but mutations in the piRNA pathway proteins result in ectopic expression in early oocytes as well as defects in germline development [6, 59]. Other *An. gambiae* genes in the “reproduction and development” cluster have orthologs in *D. melanogaster* where they have been experimentally linked to germline development and maintenance, spermatid development, and oogenesis (Additional file 16: Table S9). Another gene from this cluster, AGAP000561, is an ortholog of the *D. melanogaster* Kinesin heavy chain (FBgn0001308), which plays a role in *oskar* mRNA localization to the pole plasm [60].

**Fig. 9:**
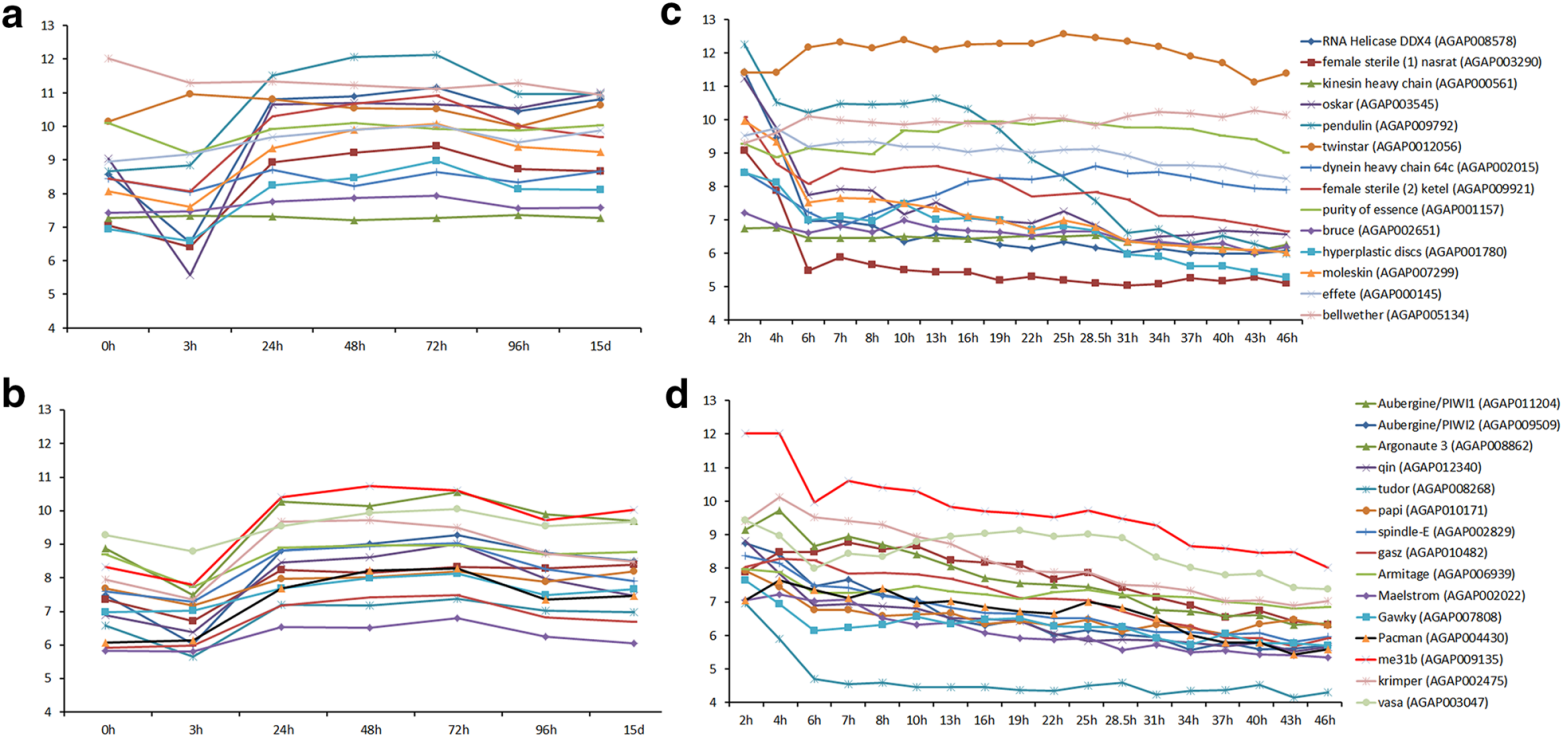
Trends in gene expression of 14 “reproduction and development” cluster genes and *An. gambiae* genes involved in the piRNA pathway in relation to time post-blood feeding (**a**, **b**) and embryonic development (**c**, **d**)

We further analyzed expression profiles for the group of 14 reproduction- and development-related genes. These expression profiles, available in the Expression Browser [54] through VectorBase [61] were analyzed to potentially discover common trends associated with these 14 genes that may provide insight into the production pattern of these piRNAs (Fig. 9). Nine of the 14 genes exhibited a down-regulation at 3 h after blood feeding. The 3-h time point is likely when metabolism functions are shifted toward blood digestion [62], making this part of the gonotrophic cycle a critical stage for proper egg formation. Twelve of 14 genes showed an up-regulation 24 h after blood feeding indicating their possible role in ovarian development (Fig. 9a). This expression pattern was similar to that of many genes involved in the piRNA pathway: 11 of 15 genes were down-regulated at 3 h after blood feeding, and all 15 genes were up-regulated at 24 h after blood feeding (Fig. 9b). Our piRNA library is derived from ovaries dissected at 24 h after blood feeding, and the high numbers of piRNAs mapped to the reproduction- and development-related genes suggest interplay between piRNA production and gene transcription (see “Discussion”). Furthermore, 11 of 14 genes from the “reproduction and development” cluster exhibited a pronounced decline in expression between 2 and 6 h of embryonic development, followed by a relatively constant level of expression afterward (Fig. 9c). Similarly, expression of 10 of 15 genes involved in the piRNA pathway also declined between 2 and 6 h of embryonic development (Fig. 9d). The transcripts of the 15 piRNA pathway genes themselves can produce piRNAs as well, but the amount is approximately 2.5 times less than the amount of piRNAs derived from transcripts of the “reproduction and development” cluster genes (Additional file 17: Table S10). This suggests that the piRNA production from the “reproduction and development” cluster genes is rather specific, and might have a functional role in development (see “Discussion”).

## Discussion

A role of the piRNA pathway in the TE mobilization control and in germline development has been demonstrated for *D. melanogaster* [1]. *Anopheles gambiae* and *D. melanogaster* delineated approximately 260 million years ago (Fig. 1), and the degree of conservation of piRNA functions between these two species is an open question. Moreover, Dipteran species vary greatly in genome size and the pattern of genomic distribution of TEs: from a highly compartmentalized, small genome (144 Mb) in *D. melanogaster* with TEs concentrated in pericentromeric heterochromatin [34], to an intermediate genome size (273 Mb) in *An. gambiae* [33], to a large genome (1310 Mb) in *Ae. aegypti* with homogeneously high TE coverage [35, 38]. How does the organization of the piRNA pathway change with varying genome size and repeat landscape? By studying piRNAs in *An. gambiae*, we uncovered conserved and diverse features of the piRNA pathway across Dipteran species and gained new insights into its role in the regulation of the reproductive processes in the African malaria vector.

### The *An. gambiae* piRNA pathway chiefly defends against mobilization of transposable elements

Employing the “overlap” method (see above), we found that almost 40 % of the piRNAs mapped to TEs in *An. gambiae*, which is almost twice higher than 23.6 % determined by the traditional “consensus” method, but still twice less than the 81 % of piRNAs mapped to TEs by the “overlap” approach in *D. melanogaster.* This difference may also suggest that the mosquito has a larger proportion of non-transposon-derived piRNAs than the fruit fly. Alternatively, due to the different relative abundance of TE classes, the secondary quantity of LTR retrotransposons in *An. gambiae* may, at least partially, explain the lower percentage of TE-mapping piRNAs.

We show that TE families with the largest number of derived piRNAs do not correspond to the most prevalent TE families in the mosquito genome. DNA transposons and non-LTR retrotransposons are the most abundant subclasses of TEs in *An. gambiae* (Fig. 3b, d), yet LTR retrotransposons are responsible for the origin of 60 % of the TE-derived piRNAs identified by the “overlap” method (Fig. 3f). A study of *An. gambiae* TEs concluded that elements from the major families of LTR elements (BEL/Pao, copia, and gypsy) correspond to putatively active elements [63]. Many of the DNA transposons are highly degraded, with only Mariner, P elements, and MITE*s* showing full-length sequences that can be associated with recent transposition [64]. Although some elements may be prevalent within the genome, increased activity within the ping-pong amplification loop, TE mRNA transcript abundance, and presence within clusters have all been found to be important in determining piRNA abundance [65]. Moreover, as in *Drosophila* [65], the most transpositionally active TE families may not be necessarily the TEs that give the most abundant piRNAs. The preference toward specific classes of TEs may help to explain how other classes of TEs (DNA transposons) have become more pervasive in the mosquito genome.

### Peculiar aspects of genomic distribution and organization of piRNA clusters in *An. gambiae*

In *D. melanogaster,* the piRNA clusters are predominantly heterochromatic, and found within pericentromeric regions [2]. Prior to this study, it was unclear if piRNA clusters are mainly located in pericentromeric heterochromatin in other Dipteran species. Using an approach that was standardized between *D. melanogaster* and *An. gambiae*, we identified clear differences in the genomic location and composition of the piRNA clusters among Dipteran species. piRNA clusters were concentrated in the pericentromeric heterochromatin of *D. melanogaster*, while an increasing number of clusters were found in the intercalary heterochromatin and euchromatin of *An. gambiae*. *Aedes aegypti* serves as the most extreme example of piRNA cluster shifting, having major clusters mainly located in euchromatic loci. The fact that we find no identified piRNA clusters in the compact intercalary heterochromatin of *An. gambiae* influences the overall findings from this study. Our data suggest that a genomic locus being a region of heterochromatin, *i.e.,* low abundance of genes and high repeat content, is not sufficient for containing piRNA clusters, at least in *An. gambiae*.

We hypothesize that as the genome size decreases in insects, the heterochromatin location becomes more restricted to the pericentromeric regions, and TEs, concordantly, become concentrated within the heterochromatin. Similarly, as the genome size increases, heterochromatin and TEs spread into new chromosomal regions. The expansion of piRNA cluster location from pericentromeric regions follows the shift in TE location from pericentromeric heterochromatin to intercalary heterochromatin and to euchromatin. This shifting genomic distribution results in piRNA clusters with attributes more closely resembling euchromatin than heterochromatin. Change in cluster composition may change the pattern of TE and gene regulation [66]. In the *D. melanogaster* genome with well-compartmentalized TE locations, loci in pericentromeric heterochromatin are responsible for production of 41.6 % of piRNAs (Additional file 9: Table S4). piRNA clusters require the H3K9me3 repressive histone mark to be transcribed and to silence TEs in fruit flies [67]. It has been proposed that Piwi binds to heterochromatin protein 1 (HP1), which in turn recruits Su(var)3-9 to add the H3K9me3 marks [68]. Such a positive feedback loop creates conditions for piRNA cluster transcription and for stabilizing the heterochromatin. Whereas in the *An. gambiae* genome with a high TE density not confined to pericentromeric regions, only 25 % of piRNAs are produced by pericentromeric heterochromatin. We propose that the organization of the majority of piRNA clusters in the malaria mosquito is more similar to that in silkworm than in fruit fly. The total estimated genome size of *Bombyx mori* is 428.7 Mb [69], which is 3.6 and 1.54 times larger than that of fruit fly and malaria mosquito, respectively. Silkworm telomeres have telomere-specific transposons and large piRNA clusters marked with heterochromatin histone marks H3K9me2 and H3K9me3. However, 965 piRNA clusters, which are located outside the telomeres, lack the heterochromatin marks, but have euchromatin marks H3K4me2, H3K4me3, and H3K9ac [70].

Heterochromatic piRNA clusters in *D. melanogaster* are enriched in the HP1 homolog Rhino that co-localizes with UAP56, which binds to piRNA precursors [48, 71]. *An. gambiae* lacks the Rhino ortholog (according to FlyBase [72], http://flybase.org/reports/FBgn0004400.html) and has a higher abundance of unidirectional euchromatic piRNA clusters (~55 %) than does *D. melanogaster*, where a majority of piRNA clusters (~78 %) are bidirectional (Fig. 5). Future studies are needed to understand the nature of the majority of piRNA clusters in the mosquito. The dispersal of piRNA clusters in euchromatin may lead to more potential cases of *cis*-regulation, in which a TE or gene is regulated by a cluster located nearby, instead of *trans*-regulation, where the regulation is provided by a pericentromeric heterochromatic cluster. Finally, manipulating the piRNA function in species with spread out clusters is likely more challenging because of the greater decentralization of the piRNA pathway.

### Novel insights from *An. gambiae* genes enriched in sense piRNAs

We identified a large proportion of sequenced piRNAs (11 %) that is associated with protein-coding genes in *An. gambiae.* In addition, 58.3 % of piRNAs are mapped to unannotated sequences, a portion of which may represent novel protein-coding genes, TEs, or long non-coding RNA genes (Fig. 2b). A majority of the piRNAs that mapped to protein-coding genes in *An. gambiae* was found in the sense orientation with respect to the transcript. Recent studies provide insights into how these genic piRNAs might be produced. Artificial insertion of a sequence within the 3′ UTR of a piRNA-producing gene resulted in the formation of piRNAs derived from the novel sequence [73]. Genes with TE sequences, considered “trigger piRNAs” within the transcript, can initiate secondary 3′-directed piRNA biogenesis that result in “responder” piRNAs that are in the same orientation as the genic mRNA [74–76]. These piRNAs lack partner piRNAs, and they are most likely generated outside of the ping-pong amplification loop, unless they target other, yet undiscovered genes. For example, piRNAs produced by *traffic jam* are loaded onto Piwi to silence specific target gene *fasciclin 3* in *D. melanogaster* [19].

Can the sense strand bias give us a clue about a possible mechanism of gene regulation via piRNAs? For example, the *An. gambiaeoskar* had 398 genome-unique sense reads and no antisense reads (24–29 nt, zero mismatches), meaning that all these piRNAs derive from the *oskar* primary transcript or mRNA. Could a high level of piRNA production lead to a depletion of mRNAs and, thus, to reduced levels of the protein? This would be possible if the piRNA and mRNA production pathways compete with each other by using the same primary transcripts as a source for either piRNAs or mRNAs. This also would be possible if piRNAs are produced from mRNAs. Because the levels of piRNA production are likely regulated by the PIWI proteins, we investigated expression profiles of genes involved in the piRNA pathway in *An. gambiae* blood feeding and embryonic development experiments. Indeed, we found a good correspondence between the patterns of expression of genes from the “reproduction and development” cluster and genes from the piRNA pathway (Fig. 9). A possible reason for these parallel trends of expression is that the piRNA machinery processes primary gene transcripts or mRNAs into piRNAs. For example, when up-regulation of “reproduction and development” genes creates a high abundance of transcripts, the parallel up-regulation of the PIWI genes would process some of these gene transcripts into piRNAs, thus reducing the amount of mRNA. Otherwise, these reproductive genes and piRNA pathway genes could be regulated by the same factors. The idea that piRNAs can be produced from primary transcripts or from mRNAs is supported by a correlation analysis, which found that more piRNAs are produced by more highly expressed genic transcripts [77]. Nevertheless, many highly expressed transcripts do not produce piRNAs, suggesting restricted access of substrates to the piRNA biogenesis machinery. Furthermore, it has been shown that the protein level of the *traffic jam*, whose 3′ UTR generates abundant sense piRNAs, is upregulated in *Drosophilapiwi* mutants [20]. Studying the functional role of these piRNAs would require knocking down the piRNA production from all these specific genes without changing their protein expression.

If piRNAs do indeed play a role in regulation of mRNA abundance, it may be to aid in the transition from maternally deposited mRNA to zygotically transcribed mRNA levels. Many of the genes from the “reproduction and development” cluster and from the piRNA pathway exhibited a pronounced decline in expression between 2 and 6 h of embryonic development (Fig. 9). As described earlier, zygotic transcription occurs as early as 2–3 h into embryogenesis [78]. Prior to this, large quantities of mRNAs are maternally deposited into the embryo. Since we see such a marked decrease in the abundance of mRNAs within this cluster of reproductive development genes, it may be possible that the processing of mRNAs into piRNAs is an underlying cause of the decline of maternally deposited mRNAs.

Another gene regulation mechanism is transcript suppression, which occurs in many *D. melanogaster* piRNA clusters that exhibit heterochromatic characteristics, including the epigenetic marks H3K9me3 and HP1 [67, 79]. In *D. melanogaster*, maternally deposited embryonic piRNAs are replaced by TE-derived siRNAs [80], which are posited to aid in the spread of heterochromatin formation through a shift in recruitment of HP1 and related proteins, contributing to gene silencing. If similar epigenetic modifications occur in regions typically devoid of heterochromatin marks, it is possible that the recruitment of heterochromatic proteins in or near these euchromatic regions containing protein-coding genes results in repressed transcription through heterochromatinization. It is unclear if the chromatin state itself is what allows piRNA production [48]. Given the nuclear localization of Piwi, it is conceivable that the Piwi-piRNA complex could associate with the piRNA-producing genes, providing an epigenetic transcriptional regulation through histone modification [43].

## Conclusion

Our study demonstrates that the distribution and organization of piRNA clusters observed in fruit flies may not be conserved in other Dipteran species. The more decentralized genomic location of piRNA clusters in *An. gambiae* and *Ae. aegypti* compared with *D. melanogaster* could potentially cause the generation of a larger proportion of non-TE-derived piRNAs. The large number of piRNAs that originate from regions other than pericentromeric heterochromatin suggests that their roles may be more diverse in mosquitoes than in *D. melanogaster*. Moreover, *An. gambiae* has a higher abundance of unidirectional euchromatic piRNA clusters than *D. melanogaster* does, which points to important differences between the piRNA machineries of the two species. Identification of the large pool of piRNAs produced by genes involved in reproduction and development indicates that the piRNA pathway may play a role in reproductive processes in the malaria vector. Future research will lead to understanding the epigenetic mechanisms of how these piRNAs regulate gene expression and affect germline and embryonic development. Our study also suggests that LTR retrotransposons have a distinct capacity to produce piRNAs that may be linked to higher transcription rate, higher frequency of insertion into piRNA clusters and/or higher capacity to enter the ping-pong amplification cycle. The study of piRNA production from various classes of TEs may offer understanding of the potential ability of some TEs to escape piRNA repression.

## Methods

### Total RNA isolation and small RNA library construction

Christopher’s Stage III ovaries were dissected from 25-h gravid females of the Mali strain (M form) of *An. gambiae* obtained from the Malaria Research and Reference Reagent Resource Center (MR4). Isolated ovaries were preserved in Trizol to prevent RNA degradation. Total RNA was extracted from ovaries of approximately 40–50 individual mosquitoes. RNA was precipitated using a standard phenol–chloroform extraction, solubilized in RNase-free water and stored at −80 °C. Total RNA was isolated, and Illumina sequencing was performed by Fasteris, Inc. on the RNAs ranging from 18 to 32 nucleotides in length. A library containing RNAs ranging in size from 18 to 32 nt was created from a single RNA sample. The library had a sharp peak at 22 nt, with a secondary broad peak ranging from 24 to 30 nt that apexed at 26–27 nt.

### Small RNA library modification

A total of three libraries were generated for subsequent piRNA analysis. Libraries corresponded to two specific factors: read count within the library (collapsed vs. non-collapsed) and instances of a read found within the *An. gambiae* genome (unique vs. non-unique). The short-read mapper NucBase was run to determine the number of times each RNA sequence mapped within the *An. gambiae* genome [81]. The initial sequenced library, consisting of 22,569,568 total non-unique, non-collapsed reads from 24 to 29 nt, was used for mapping piRNAs to TEs. A second library of 6,805,309 RNAs containing non-collapsed, unique reads (i.e., redundant reads that map to a single genomic locus) was used for gene analysis. A final library consisting of 568,080 collapsed, unique reads (i.e., non-redundant, single genome-mappers) was used for piRNA cluster identification.

### Mapping small RNA reads to reference genome sequences

For initial inter-specific comparative assessment of NCNU piRNA libraries, the small RNA reads, 24–29 nt long, were mapped to the AgamP4, AaegL3, and Dmel Release 6.04 genome assemblies for *An. gambiae*, *Ae. aegypti*, and *D. melanogaster,* respectively, allowing zero mismatches (Fig. 2). For the *An. gambiae* analysis, we used the small RNA library from ovaries that contains 16,691,820 genome-mapping piRNAs. For the *D. melanogaster* analysis, we used the small RNA library from ovaries (sample GSM872307) that contains 7,465,629 genome-mapping piRNAs. For the *Ae. aegypti* analysis, we used the small RNA library from whole adults [22] that contains 388,136 genome-mapping piRNAs.

The resulting genome-mappers were run through size analysis. Annotation of small RNAs was done by mapping the reads on data downloaded from VectorBase [61], FlyBase [72], and MirBase [82] allowing zero mismatches with the exception of TEs being downloaded from RepBase [42] and mapped allowing 0–3 mismatches. The AgamP4 assembly of the *An. gambiae* PEST genome was used as the reference genome for localization by the short-read mapper, NucBase [81]. NucBase counts the number of times a specific RNA sequence maps to the reference genome, allowing the user to filter out all repetitive sequences from the library. We ran NucBase using zero mismatches. As mentioned previously, unique mapping piRNAs were parsed into a secondary, final library that was used to discover the location of piRNA clusters.

BowTie2 was used to map the small RNAs to both TEs and gene transcripts. Default settings were used within the program, with parameters including end-to-end mode, a minimum seed length of 22 nt, and a mismatch penalty of 6. Up to three mismatches were allowed to account for sequence degradation. TEs for the subphylum Hexapoda, available on RepBase [42], were used for all repeat masking and piRNA localization. The AgamP4.1 transcript file was used for identifying piRNAs mapping to genes in *An. gambiae*.

### Identification of piRNA clusters

To identify the genomic loci responsible for piRNA generation in *An. gambiae*, data were restricted to sequences that mapped uniquely across the reference genome. All 24–29 nt reads were initially mapped to the *An. gambiae* PEST AGamP4 genome assembly (without repeat masking) using NucBase [81], a short-read mapper designed to align short sequence reads from large nucleic acid databases to genomes or input sequences. After the initial mapping, genome-unique reads, i.e., mapping only once in the *An. gambiae* genome, were retained, and reads present more than once in the library were reduced to one occurrence, resulting in a non-duplicate (collapsed) library of unique mapping reads that was then re-plotted with NucBase. The final mapping run resulted in 568,080 small RNA sequences that mapped to the five chromosomal arms (2R, 2L, 3R, 3L, and X), as well as to the non-assembled chromosome UNKN.

Initial short-read mapping was completed on 5-kb windows for each chromosome arm of the *An. gambiae* AgamP4 assembly. A Perl script was written to identify consecutive windows containing a minimum of ten unique piRNAs that held a single constraint. This constraint allowed the inclusion of gaps of up to four contiguous windows (or 20 kb) containing less than ten piRNAs each that could continue the cluster. The ten piRNA cutoff value used in the *Ae. aegypti* study [22] was necessary to improve cluster detection when working with such a large dataset. To be considered a cluster, 0.05 % of the subset of 568,080 uniquely mapping piRNAs, or 284 piRNAs, was the minimum total piRNAs within a given region.

We ran our piRNA cluster analysis pipeline using the *w*^*1118*^ piRNA library (sample GSM327620) [44] on the Dmel_R6.04 release of the *D. melanogaster* [34] genome assembly (without repeat masking), using the same methodology as for *An. gambiae*. We used the unique 24–29 nt sequences as the input piRNA library and excluded auxiliary sequence scaffolds from cluster identification because heterochromatin is better assembled in *D. melanogaster* than in *An. gambiae*. A threshold of 101 piRNAs, 0.05 % of the 202,533 unique piRNA mappers used in the mapping analysis, was set for the minimum number of mappers within consecutive windows that constituted a cluster. Cytological positions of piRNA clusters were determined using the “estimated cytological band” track in the FlyBase genome browser [72].

### TE, gene ontology, and expression data analysis

The TE identification was performed by using the RepeatMasker tool to search against all available annotated elements. We elected to use subphylum Hexapoda TEs when performing RepeatMasker searches, as the complete annotation of TEs in *An. gambiae* is still lacking. For “overlap” identification, the genome was masked using the Hexapoda subphylum from RepBase [42] in RepeatMasker [45]. Masked regions (correspondent to TEs) were then mapped by piRNA sequences to identify “overlapping” piRNA-TE sequences and positions, while allowing no mismatches. These positions were then marked to identify the associated TE family/class. We used the −k = 1 option in BowTie2, which reports a single valid alignment for each read in the library. This setting allows the report to identify mapping to TE sequences without over-reporting hits from identical, repetitive sequences. We tested using seed length (allowing for the piRNA to extend past the repeat-masked sequence), but saw minor differences using a seed length of 16 and not using a specific seed. The aligner was run in end-to-end alignment mode. Repeat-masked regions representing TEs were all concatenated into a single file, and piRNA reads were aligned to these sequences allowing no mismatches.

piRNAs were localized to gene exon DNA sequences extracted using BioMART [83–85]. Gene ontology terms for piRNA-mapped genes were identified using DAVID v6.7 [56, 57]. The default settings in DAVID were used with the addition of Bonferroni correction of P values for identification of significant GO terms. Using DAVID’s categorization of gene functions, subsets of genes were identified that may be important within the piRNA pathway. Specifically, the subset of genes implicated in reproduction and development was used when looking at expression assays. Differential expression values associated with those 14 genes were further examined to identify potential trends between the genes that could provide insight into the relationship between the gene and the piRNA pathway.

## Availability of supporting data

The BioProject PRJNA278159 files are available from the NCBI SRA database (accession number: SRX966734).

## Additional files

10.1186/s13072-015-0041-5 Size distribution of all genome-mapping small RNAs sequenced from the *An. gambiae* ovaries.
10.1186/s13072-015-0041-5 piRNA library definitions and sizes for *An. gambiae* and *D. melanogaster.*

10.1186/s13072-015-0041-5 The piRNA pool derived from TE sequences in *D. melanogaster* and *An. gambiae.*

10.1186/s13072-015-0041-5 TE-derived piRNA direction bias suggests a ping-pong-like mechanism. % of TE-derived piRNAs in relation to TE orientation is identified by the “overlap” method. % sense indicates the piRNA is in the same orientation as the coding strand of the consensus TE, while % antisense specifies piRNAs complementary to this strand. w/o UN, without chromosome UNKN. w/ UN, with chromosome UNKN.
10.1186/s13072-015-0041-5Distribution and composition of piRNA clusters in the *An. gambiae* genome.
10.1186/s13072-015-0041-5 Structure of the top three piRNA clusters in *An. gambiae*. **A)** Unidirectional euchromatin cluster. **B)** Bidirectional intercalary heterochromatin cluster. **C)** Bidirectional pericentromeric heterochromatin cluster. piRNA mapping (blue for sense reads and red for antisense reads) across the span of an individual cluster. Y axis indicates number of unique piRNAs at a given position (X axis). Repeat-masked TEs are identified by black boxes at the horizontal (X) axis.
10.1186/s13072-015-0041-5 Distribution and composition of piRNA clusters in the *D. melanogaster* genome.
10.1186/s13072-015-0041-5 Distribution of TEs and clusters of collapsed, unique piRNAs in the chromosome-based genome assembly of *An. gambiae*. TE percentage across each chromosome arm is broken down by 25-kb windows. Heterochromatin is shown by dark red. Number of mapped 24-29-nt unique piRNAs is shown along chromosomes in 25-kb windows. Clusters and their respective ranks based on piRNA density are shown in light red.
10.1186/s13072-015-0041-5 Distribution and composition of top 30 piRNA clusters in the *Ae. aegypti* genome.
10.1186/s13072-015-0041-5 Distribution of TEs and clusters of collapsed, unique piRNAs in the chromosome-based genome assembly of *D. melanogaster*. TE percentage across each chromosome arm is broken down by 25-kb windows. Heterochromatin is shown by dark red. Number of mapped 24-29-nt unique piRNAs is shown along chromosomes in 25-kb windows. Clusters and their respective ranks based on piRNA density are shown in light red.
10.1186/s13072-015-0041-5 Chromosomal distribution of 24 top piRNA clusters in *Ae. aegypti*. Clusters in their respective ranks based on piRNA density are shown in blue.
10.1186/s13072-015-0041-5 Peaks of siRNAs and piRNAs mapped to gene transcript in *An. gambiae*. **A)** Size distribution of small RNAs mapped to all transcripts without AGAP003387. **B)** A high peak of siRNAs mapped to the AGAP001627 transcript. **C)** Peaks of siRNAs and piRNAs mapped to the AGAP001754 transcript.
10.1186/s13072-015-0041-5 piRNAs mapping to TEs within genes (0-3 mismatches) in *An. gambiae.*

10.1186/s13072-015-0041-5 TE family distribution of genic TE-mapping piRNAs in *An. gambiae*.
10.1186/s13072-015-0041-5 GO annotation clusters of genes with high piRNA mapping in *An. gambiae*.
10.1186/s13072-015-0041-5 GO annotation of genes in the “reproduction and development” cluster in *An. gambiae.*

10.1186/s13072-015-0041-5 piRNA mapping to the piRNA pathway genes and “reproduction and development” cluster genes in *An. gambiae*.

## Abbreviations

Ago3: Argonaute 3
Aub: Aubergine
FAT: functional annotation terms
GO: gene ontology
H3K9me2: dimethylated histone H3 lysine 9
H3K9me3: trimethylated histone H3 lysine 9
H3K4me2: dimethylated histone H3 lysine 4
H3K4me3: trimethylated histone H3 lysine 4
H3K9ac: acetylated histone H3 lysine 9
HP1: heterochromatin protein 1
LINE: long interspersed nuclear element
LTR: long terminal repeat
miRNA: microRNAs
MITE: miniature inverted-repeat transposable element
MR4: Malaria Research and Reference Reagent Resource Center
mRNA: messenger RNA
NC: non-collapsed
NU: non-unique
NCNU: non-collapsed non-unique
nt: nucleotide
piRNA: Piwi-interacting RNA
rRNA: ribosomal RNA
RISC: RNA-induced silencing complex
RPM: reads per million
SINE: short interspersed nuclear element
snRNA: small nuclear RNA
SuUR: Suppressor of Under Replication
Su(var): Suppressor of variegation
TEs: transposable elements
tRNA: transport RNA
UNKN: unknown
UTRs: untranslated regions
