# Supplementary figures and images for: Increased production of piRNAs from euchromatic clusters and genes in Anopheles gambiae compared with Drosophilamelanogaster
Source: Epigenetics Chromatin. 2015 Nov 27;8:50. doi: 10.1186/s13072-015-0041-5 (PMC4662822; doi:10.1186/s13072-015-0041-5)

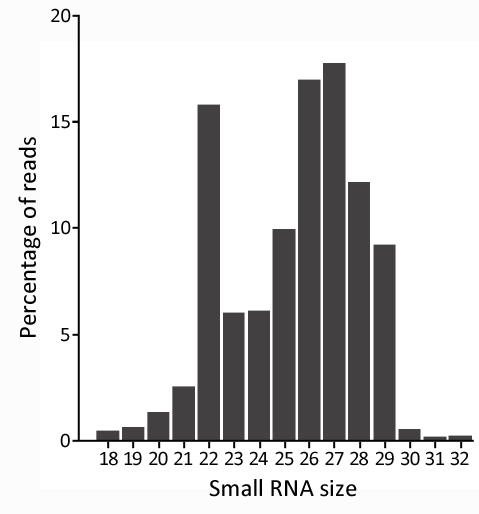

Supplement: Supplementary file 1 — 10.1186/s13072-015-0041-5 Size distribution of all genome-mapping small RNAs sequenced from the An. gambiae ovaries. [file 13072_2015_41_MOESM1_ESM.tif]

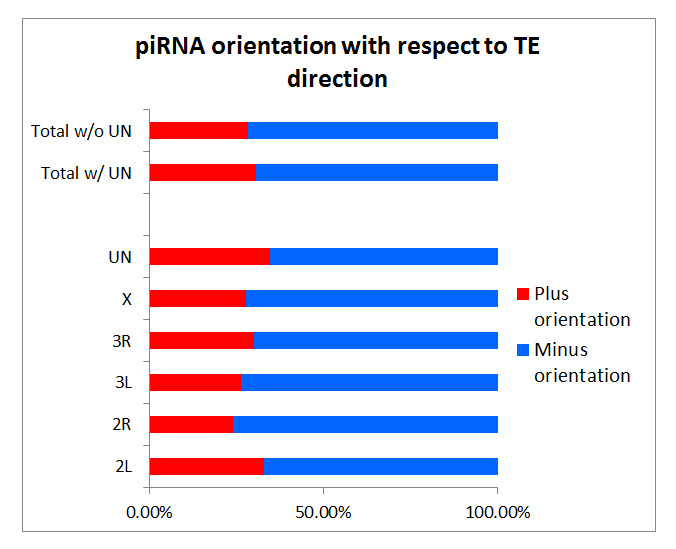

Supplement: Supplementary file 4 — 10.1186/s13072-015-0041-5 TE-derived piRNA direction bias suggests a ping-pong-like mechanism. % of TE-derived piRNAs in relation to TE orientation is identified by the “overlap” method. % sense indicates the piRNA is in the same orientation as the coding strand of the consensus TE, while % antisense specifies piRNAs complementary to this strand. w/o UN, without chromosome UNKN. w/ UN, with chromosome UNKN. [file 13072_2015_41_MOESM4_ESM.tif]

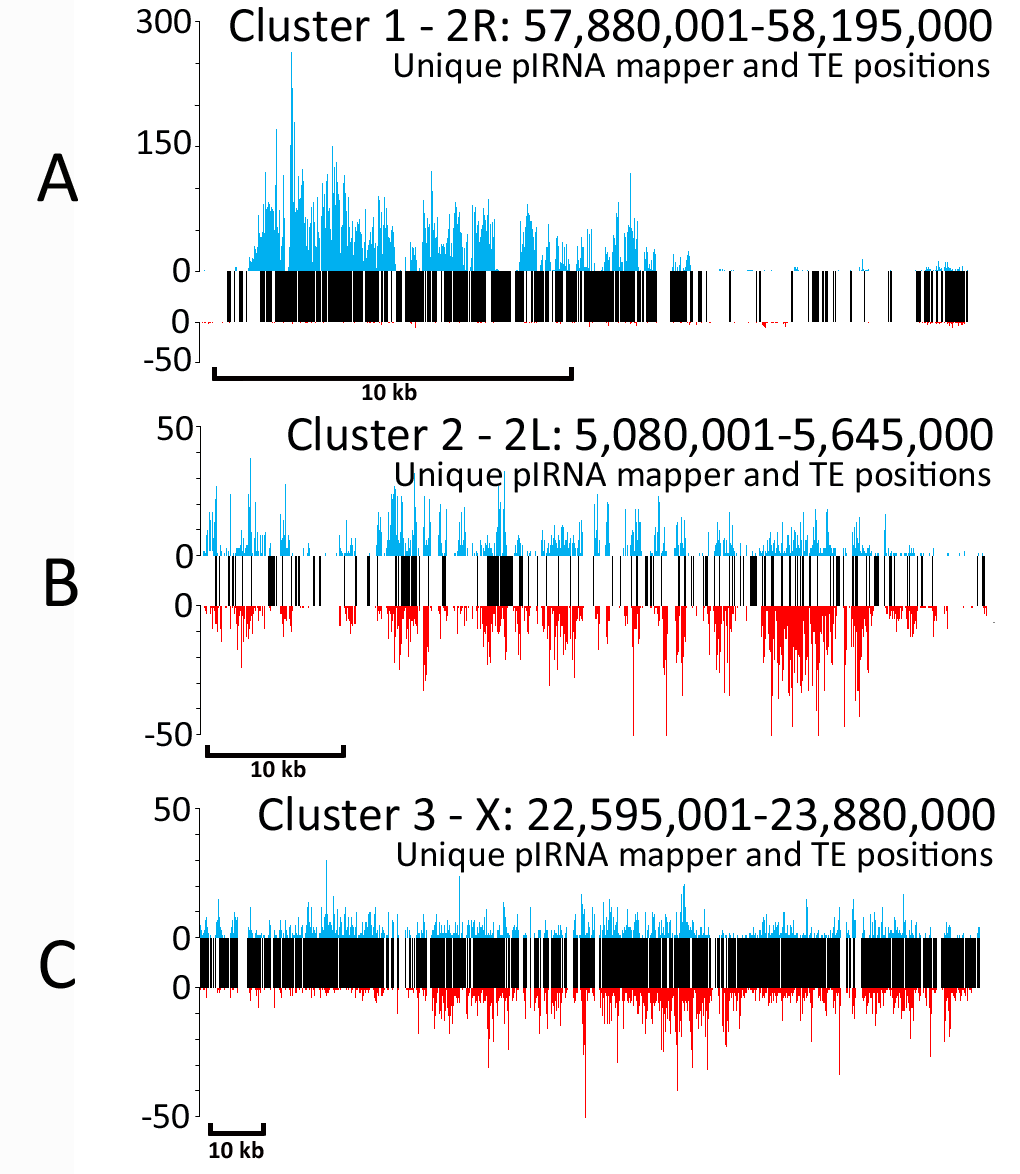

Supplement: Supplementary file 6 — 10.1186/s13072-015-0041-5 Structure of the top three piRNA clusters in An. gambiae. A) Unidirectional euchromatin cluster. B) Bidirectional intercalary heterochromatin cluster. C) Bidirectional pericentromeric heterochromatin cluster. piRNA mapping (blue for sense reads and red for antisense reads) across the span of an individual cluster. Y axis indicates number of unique piRNAs at a given position (X axis). Repeat-masked TEs are identified by black boxes at the horizontal (X) axis. [file 13072_2015_41_MOESM6_ESM.tif]

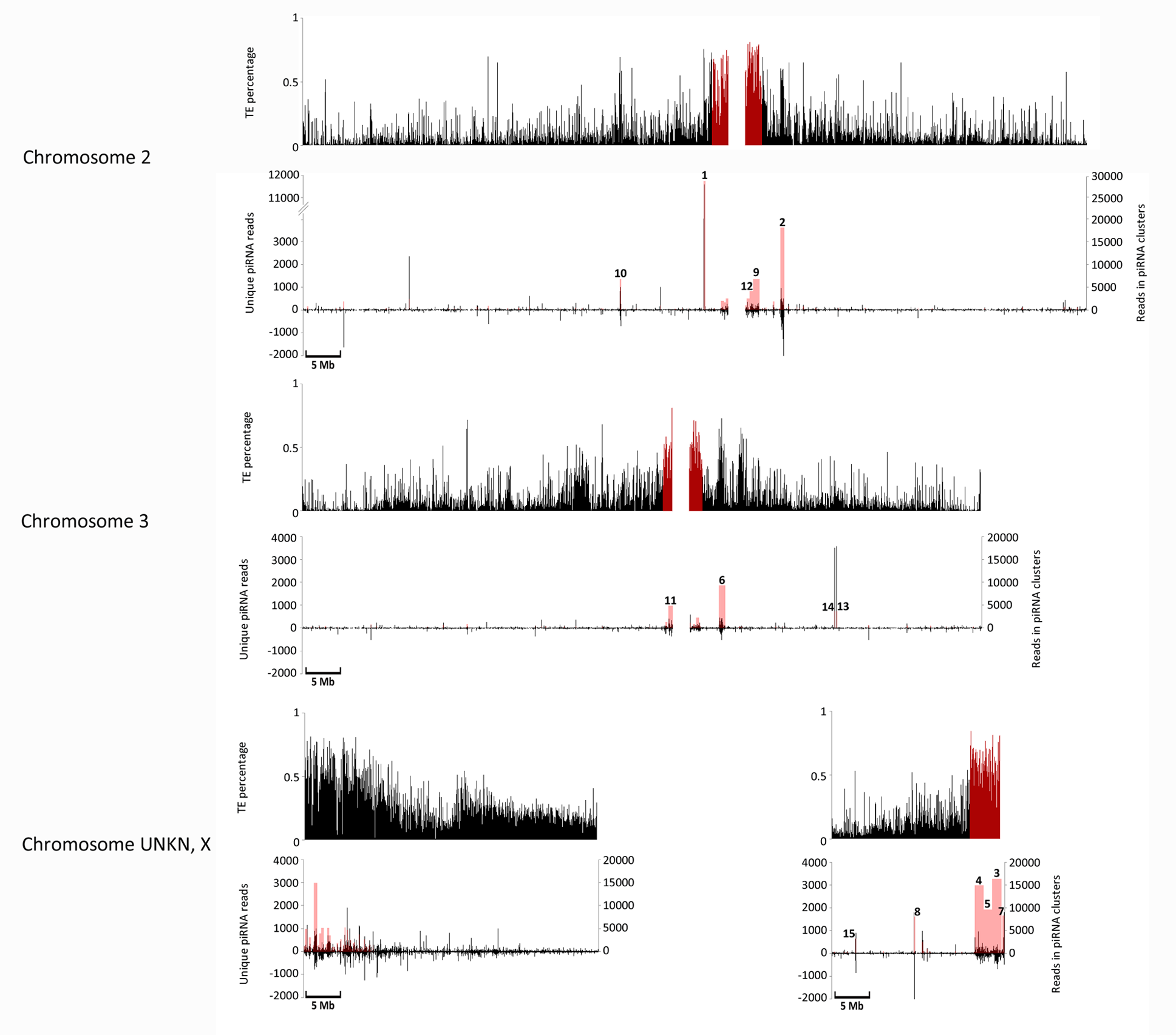

Supplement: Supplementary file 8 — 10.1186/s13072-015-0041-5 Distribution of TEs and clusters of collapsed, unique piRNAs in the chromosome-based genome assembly of An. gambiae. TE percentage across each chromosome arm is broken down by 25-kb windows. Heterochromatin is shown by dark red. Number of mapped 24-29-nt unique piRNAs is shown along chromosomes in 25-kb windows. Clusters and their respective ranks based on piRNA density are shown in light red. [file 13072_2015_41_MOESM8_ESM.tif]

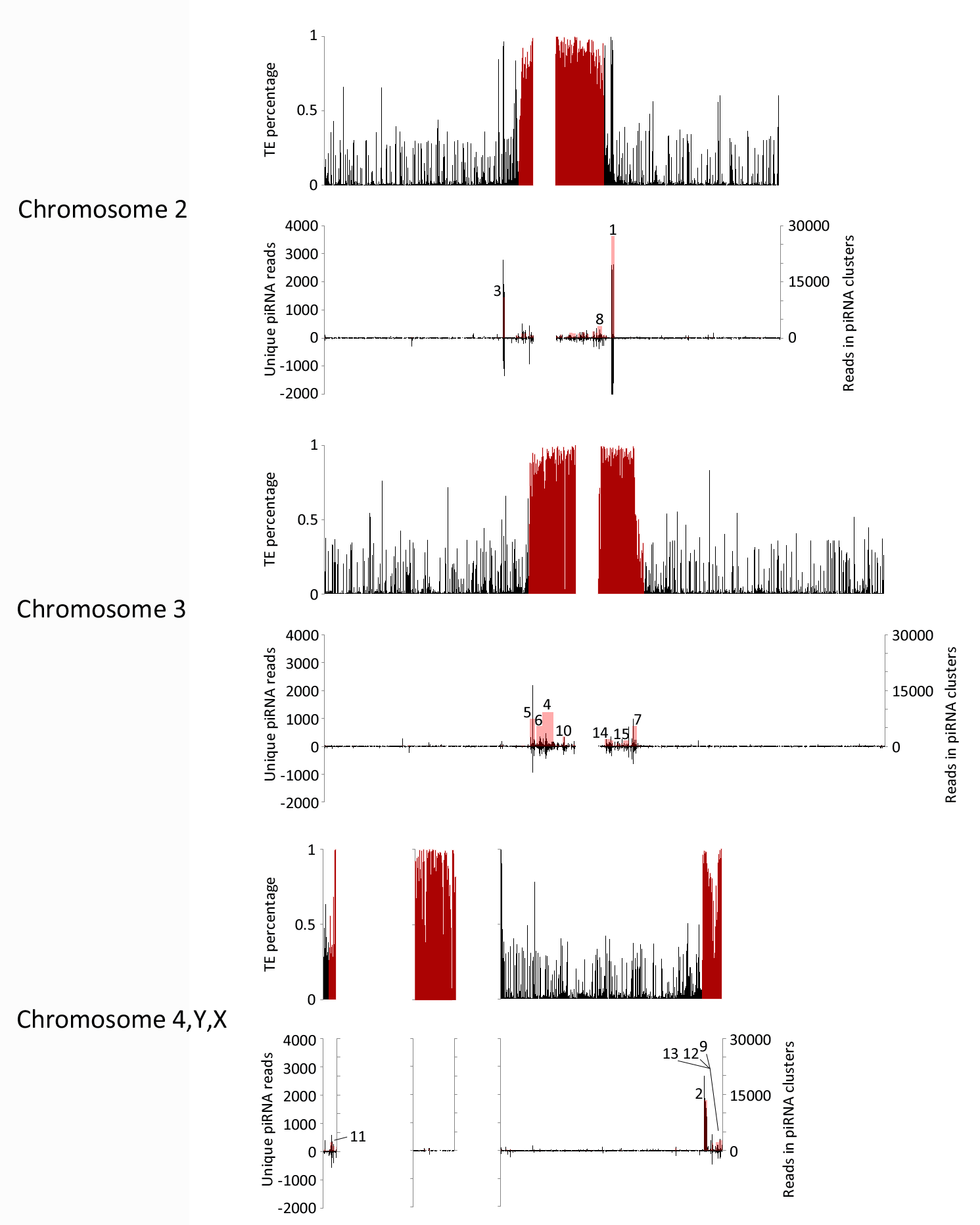

Supplement: Supplementary file 10 — 10.1186/s13072-015-0041-5 Distribution of TEs and clusters of collapsed, unique piRNAs in the chromosome-based genome assembly of D. melanogaster. TE percentage across each chromosome arm is broken down by 25-kb windows. Heterochromatin is shown by dark red. Number of mapped 24-29-nt unique piRNAs is shown along chromosomes in 25-kb windows. Clusters and their respective ranks based on piRNA density are shown in light red. [file 13072_2015_41_MOESM10_ESM.tif]

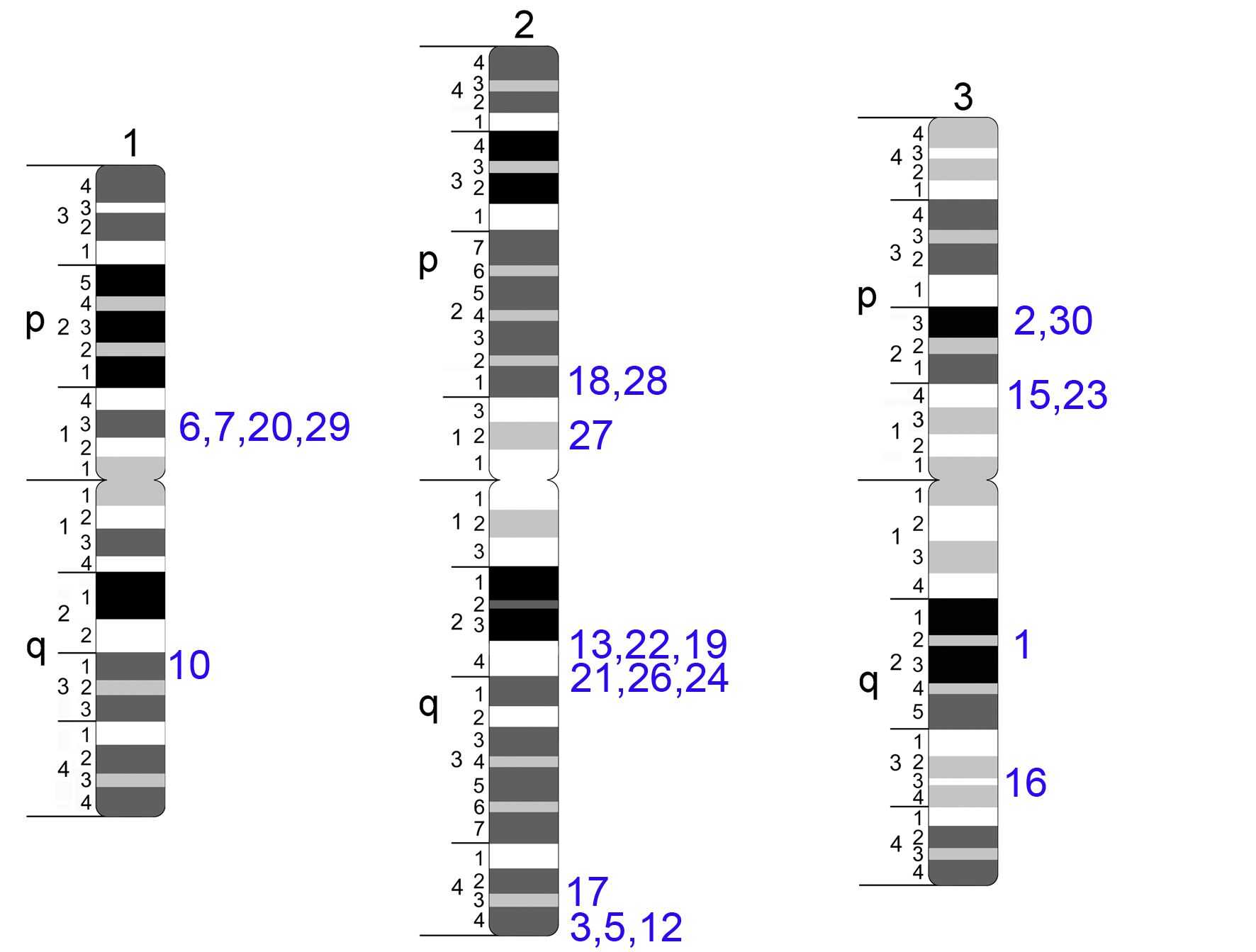

Supplement: Supplementary file 11 — 10.1186/s13072-015-0041-5 Chromosomal distribution of 24 top piRNA clusters in Ae. aegypti. Clusters in their respective ranks based on piRNA density are shown in blue. [file 13072_2015_41_MOESM11_ESM.tif]

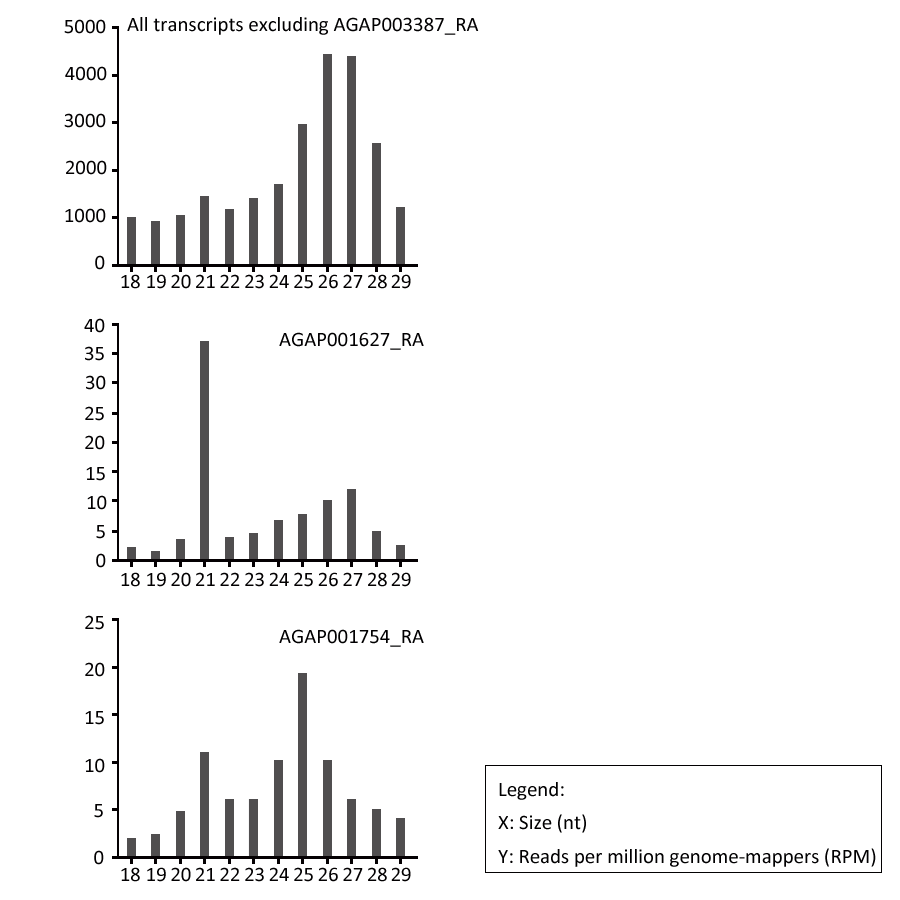

Supplement: Supplementary file 12 — 10.1186/s13072-015-0041-5 Peaks of siRNAs and piRNAs mapped to gene transcript in An. gambiae. A) Size distribution of small RNAs mapped to all transcripts without AGAP003387. B) A high peak of siRNAs mapped to the AGAP001627 transcript. C) Peaks of siRNAs and piRNAs mapped to the AGAP001754 transcript. [file 13072_2015_41_MOESM12_ESM.tif]
